# circ-0001875 downregulation is associated with M1 macrophage activation and lung inflammation in severe asthma

**DOI:** 10.3389/fimmu.2025.1601272

**Published:** 2025-06-30

**Authors:** Gege Liu, Jiahao Cao, Yiyan Lin, Bingyu Long, Yanyu Su, Guiqiang Qiu, Chi Jiang, Yue Wang, Xuanna Zhao, Dan Huang, Dong Wu

**Affiliations:** Department of Respiratory and Critical Care Medicine, Affiliated Hospital of Guangdong Medical University, Zhanjiang, China

**Keywords:** severe asthma, circ-0001875, miR-31-5p, SP1, M1 polarization

## Abstract

**Background:**

Asthma is a heterogeneous group of diseases. The mechanism by which dysregulated circRNAs affect severe asthma by regulating macrophage polarization remains unclear.

**Methods:**

High-throughput RNA sequencing technology was used to analyze circRNA expression in peripheral blood mononuclear cells (PBMCs) from patients with severe asthma. RT-qPCR and ELISA were used to analyze the expression of inflammatory factors in a mouse model of severe asthma induced by ovalbumin-lipopolysaccharide. The effect of circ-0001875 on macrophage activation and the underlying mechanism were analyzed by RT-qPCR, Western blot, and ELISA. Subsequently, the regulatory relationships among circ-0001875, miR-31-5p, and SP1 were examined through dual luciferase reporter gene assay, and the mechanism by which they regulate macrophage polarization was analyzed by Western blot.

**Results:**

Compared with the healthy control group, 420 circRNAs were differentially expressed in PBMCs from patients with severe asthma. Among them, circ-0001875, which was mainly expressed in the cytoplasm of monocytes, was significantly downregulated in asthmatics, especially those with severe disease. circ-0001875 overexpression inhibited M1 macrophage activation *in vitro* and alleviated lung inflammation in a mouse model of severe asthma. Mechanistically, circ-0001875 promoted SP1 translation by competitively binding to miR-31-5p, thereby reducing its inhibitory effect on SP1 translation; SP1 then inhibited M1 macrophage polarization, which is associated with severe asthma, through the NF-κB signaling pathway.

**Conclusions:**

We found that circ-0001875 plays an important role in regulating M1 macrophage polarization, which is associated with a severe pro-inflammatory response.

## 1 Introduction

Bronchial asthma is a heterogeneous group of diseases characterized by chronic inflammation of the airways involving various inflammatory cells including eosinophils, mast cells, lymphocytes, macrophages, and so on, as well as various inflammatory mediators. Its main features include chronic airway inflammation, airway hyperresponsiveness, and chronic airway remodeling over time (1, 2). Currently, approximately 300 million people worldwide suffer from asthma, posing a serious threat to global public health (3). Severe asthma is a clinically defined subgroup of asthma that frequently responds poorly to treatment with corticosteroids. Corticosteroids are currently recommended for the treatment of persistent asthma and are the preferred therapy for effectively controlling airway inflammation (4). Corticosteroid resistance is a significant challenge to the treatment of refractory asthma (5, 6).

Macrophages are the main innate immune cells in the lungs, accounting for over 70% of pulmonary immune cells (7). Upon exposure to external allergens, pulmonary macrophages are activated and participate in pro-inflammatory and anti-inflammatory processes (8). Classically activated (or M1) macrophages induced by IFN-γ, lipopolysaccharide (LPS), and granulocyte macrophage colony stimulating factor (GM-CSF) are associated with pathogen clearance and involved in the pathogen-driven innate immune response. In contrast, alternatively activated (or M2) macrophages induced by IL-4 and IL-13 are associated with tissue remodeling and cell clearance and involved in anti-inflammatory reactions (9, 10). Previous studies have shown that M1 macrophages are involved in asthma pathology. In severe asthma, macrophages adopt the M1 phenotype and produce a large amount of pro-inflammatory mediators (including TNF-α, IL-1β, IL-6, NO, etc.) that promote airway mucus secretion, exacerbate lung injury, and accelerate airway remodeling (11, 12). Our previous study showed that the nuclear factor kappa B (NF-κB) pathway is one of the main pathways that induces M1 macrophage polarization and regulates asthma-related airway inflammation and remodeling (13).

Circular RNAs (circRNAs) and microRNAs (miRNAs) are non-coding RNAs (ncRNAs) that regulate various pathological processes involving macrophages, such as macrophage polarization, airway inflammation, and airway remodeling, and are abnormally expressed in asthma (14–18). circRNAs modulate various biological processes such as macrophage polarization, immune regulation, and airway remodeling, and are involved in asthma pathology (19, 20). CircRNAs are regulatory factor for various cellular and biological processes in asthma airway smooth muscle cells, including proliferation, apoptosis, migration, and secretion of inflammatory mediators. Dysregulated circRNAs may also lead to dysfunction of bronchial epithelial cells associated with asthma, playing a critical role in T cells development and function (20). As competitive endogenous RNAs, circRNAs can act as miRNAs sponges, thereby altering the function of proteins and signaling pathways that are regulated by miRNAs (21, 22). miRNAs regulate macrophage activation during asthma progression (23, 24). miRNAs control the M1/M2 macrophage polarization balance and immune regulatory response by regulating the expression of different transcription factors (25–31). Therefore, circRNAs and miRNAs that are differentially expressed in asthma patients may participate in immune regulation, airway inflammation, and remodeling by regulating macrophage activation. However, the specific mechanisms by which dysregulated circRNAs and miRNAs affect severe asthma have not been studied.

Specific protein 1 (SP1) is a member of the zinc finger transcription factor family, which includes at least four SP transcription factors (32, 33). Previous studies have suggested that SP1 is involved in monocyte activation. SP1 can be induced and activated by LPS in THP-1, a human monocyte cell line (34, 35). SP1 also binds the enhancer or promoter region of the GM-CSF gene (35–39). As an M1 macrophage stimulating factor, GM-CSF regulates NF-κB expression (40, 41). SP1 not only binds to NF-κB, but also regulates NF-κB activation in cancer (42, 43). We previously found that SP1 expression is regulated by circRNAs that act as sponges to inhibit miRNA activity, thereby affecting the epithelial mesenchymal transition; this suggests that SP1 may be a downstream regulator of cell function and phenotype changes induced by circRNAs (44). However, whether SP1 expression is modified by dysregulated circRNAs during asthma progression, and the specific mechanisms by which SP1 participates in M1 macrophage polarization, remain unclear.

In this study, we investigated the role and regulatory mechanism of abnormally expressed circRNAs in severe asthma. We found that circ-0001875 expression levels are associated with asthma severity. Compared with healthy individuals, circ-0001875 expression is reduced in patients with severe asthma. Importantly, circ0001875 regulated M1 macrophage polarization by acting as a sponge for miR-31-5p, thereby promoting SP1 expression. We also found that the circ-0001875/miR-31-5p/SP1 axis regulates the NF-κB signaling pathway, which is involved in M1 polarization. This study reveals for the first time the mechanism by which circ-0001875 participates in asthma inflammation by regulating macrophage polarization, providing new experimental evidence for understanding the role of circRNAs in asthma. However, its potential as a clinical biomarker still needs further validation in the following areas.

## 2 Materials and methods

### 2.1 Research subject recruitment and specimen collection

All research subjects, male and female, were over 18 years old. We collected peripheral blood samples from 101 patients, including 32 in the mild asthma group, 34 in severe asthma group, and 35 in the healthy control group. The patients in the asthma groups were selected from patients who received outpatient or inpatient treatment in the Respiratory and Critical Care Department of Guangdong Medical University Affiliated Hospital from January 2020 to December 2022 and were ultimately diagnosed with asthma following the diagnostic standards in the “Guidelines for the Prevention and Treatment of Bronchial Asthma (2020 Edition)” formulated by the Asthma Group of the Respiratory Branch of the Chinese Medical Association in 2020 (45). The exclusion criteria for the asthma group were as follows (1): acute respiratory tract infection or corticosteroid treatment within 4 weeks prior to the visit; (2) underlying diseases that could have interfered with the study; (3) lack of informed consent from the patient to participate in the study. Healthy individuals for the healthy control groups were selected from patients who underwent physical examinations at the Department of Health Examination Department of Guangdong Medical University Affiliated Hospital from January 2020 to December 2022. The inclusion criteria for the healthy control group included: (1) no abnormalities detected during the routine physical examination and no history of allergic disease; (2) matching the average age and gender composition of the asthma groups; (3) informed consent obtained to participate in the study.

The clinical data and relevant examination results of the research subjects were recorded, and peripheral venous blood samples were collected (5 mL in the morning on an empty stomach) in sodium heparin vacuum tubes. Ethical approval for the study was obtained from the Ethics Committee of Guangdong Medical University Affiliated Hospital, and all experiments were carried out in strict accordance with regulations. All of the study participants provided written informed consent.

### 2.2 Cell lines

Normal lung epithelial cell (BEAS-2B), normal human bronchial epithelial cell (HBE), human embryonic kidney cell (293A), and human monocyte (THP1) lines were purchased from the cell bank of the Chinese Academy of Sciences (Shanghai, China). The cells were cultured in high-glucose DMEM complete medium (containing 10% fetal bovine serum) and RPMI 1640 complete medium (containing 10% fetal bovine serum), respectively and incubated at 37 °C with 5% CO_2_.

### 2.3 Animal model

Healthy female SPF C57BL/6 mice, 6 weeks old, weighing 16 to 18 g, were purchased from Guangdong Medical Experimental Animal Center (Guangdong, China). The mice were housed at a temperature of 20 to 24 °C with a 12/12-hour light/dark cycle and a relative humidity range of 40% to 70%. The mice were given one week to acclimate before starting the experiments. Animal ethics approval was obtained from the Quality Inspection Unit: Animal Ethical and Welfare of Affiliated Hospital of Guangdong Medical University (License No.: AHGDMU-LAC-B-202404-0020).

The experimental mice were randomly divided into four groups, each consisting of five mice: the control group (PBS), severe asthma group (ovalbumin-lipopolysaccharide [OVA-LPS]), circ-0001875 negative control with severe asthma group (OVA-LPS+con plasma), and circ-0001875 overexpression with severe asthma group (OVA-LPS+circ-0001875). The severe asthma model was established according to previous studies (46, 47). Sensitization: on Day 1 and Day 14, a sensitization solution consisting of 2.25 mg aluminum hydroxide and 20 μg OVA in 0.1 mL of PBS was intraperitoneally injected into each mouse mice. Boosting: on Day 27, 10 μg of LPS in 60 μL of solution was administered intranasally to each mouse mice. Challenge: on Days 28 to 30, 15 mL of 1% OVA in saline was administered to each mouse via atomized inhalation for 20 minutes. The mice in the control group received an equal amount of PBS.

For *in vivo* transfection with the circ-0001875 overexpression plasmid, an overexpression vector encoding circ-0001875 under the control of the macrophage-specific CD68 promoter was constructed. On Day 27, 50 μL of a transfection solution containing 8 μg of the circ-0001875 overexpression plasmid or a negative control plasmid was administered to each mouse intratracheally. The transfection mixture included Entranster ™ *In vivo* transfection reagent (Engreen, China). On Day 31, the mice were euthanized, and samples were collected.

### 2.4 RNA sequencing

Peripheral blood mononuclear cells were extracted from patients with bronchial asthma and healthy individuals, and total RNA was isolated from the cells using TRIzol Reagent (Invitrogen, CA, USA). An Agilent 2100 instrument was used to detect RNA integrity, and samples with RNA integrity values>7.0 were selected for analysis. Ribosomal RNA was removed from the samples using a RiboMinus eukaryotic assay kit (Qiao, Valencia, CA, USA). Deep sequencing of the RNA seq library was performed using an Illumina HiSeq 2000 instrument (Illumina, San Diego, CA, USA). Paired-end reads were obtained, and differentially expressed circRNAs were identified using Edger software (v3.16.5).

### 2.5 RT-qPCR

Total RNA was extracted from tissues and cells using Trizol reagent (Invitrogen) according to the manufacturer’s instructions. cDNA was synthesized using Evo M-MLV RT Premium (AG, Hunan, China), and RT-qPCR was performed on an ABI7500 (Applied Biosystems, Foster City, CA, USA) or LightCycle 480 (Roche Applied Biosystems) instrument. U6 or β-actin served as the internal controls for miRNA and mRNA, respectively. The sequences of primers were shown in **Supplementary Table S2**.

### 2.6 RNase R treatment and actinomycin D treatment

For RNase R treatment, total RNA was extracted from the cells, and 2 μg total RNA was incubated at 37°C for 15 min with 3U/μg RNase R (Epicentre Technologies Corporation, Madison, WI, USA). For ActD, cells were cultured with 2 μg/mL ActD (Beyotime, Shanghai, China) for a specific amount of time.

### 2.7 Western blot

The transfected cells were lysed in RIPA (Beyotime) containing PMSF, and the protein concentrations were quantified with BCA reagent (Beyotime). Equivalent amounts of protein were subjected to 10% SDS-PAGE and transferred to a PVDF membrane (Millipore, Billerica, MA, USA). The membrane with blocked with 5% skim milk at room temperature for 1 hour, then incubated with the primary antibody overnight with shaking at 4°C. Next, the membrane was incubated with horseradish peroxidase-labeled secondary antibodies for 1 hour. BeyoECL star (Beyotime) was used to detect the protein signals.

### 2.8 ELISA

Cell supernatants and mouse BALF were collected, and ELISA reagent kits were used to detect the concentrations of various factors in the samples. The relative concentrations of the factors were calculated based on the standard curve.

### 2.9 Extraction of PBMCs from human peripheral blood

Peripheral venous blood was collected from patients with asthma and healthy individuals, mononuclear cells were extracted using a monocyte extraction kit, and red blood cells were removed using a red blood cell lysing reagent.

### 2.10 Fluorescence *in situ* hybridization and immunofluorescence staining

The cells were fixed with 4% paraformaldehyde and then soaked in PBS containing 0.5% Triton X-100 in a confocal dish for 30 minutes. For FISH, the cells were incubated overnight at 37°C with a FITC-labeled circ-0001875 probe (GenePharma). Anti-fading mounting medium was added, DAPI staining was performed, and the cells were imaged with Olympus laser confocal microscope (Olympus Corporation, Tokyo, Japan).

### 2.11 Dual luciferase reporter assay

circ-0001875 and SP1 luciferase reporter plasmids (wild-type and mutant) were synthesized by GenePharma. Cells were seeded into a 24-well plate. When the cell density reached around 60%, the cells were transfected with the luciferase plasmids, Renilla control plasmids, and miRNA mimetics. A dual luciferase assay system (Promega) was used to detect the luciferase and Renilla fluorescence levels. The sequences of siRNAs were shown in **Supplementary Table S3**.

### 2.12 Bioinformatics analysis

The online circRNA database CircBase (https://www.circbase.org/) was used to predict target miRNAs, and their interaction sites were predicted using Circinteractome (https://circinteractome.nia.nih.gov/). TargetScan (https://www.targetscan.org/), miRbase (https://www.mirbase.org/), and miRDB (https://www.mirdb.org/) were used to predict downstream target genes of the identified miRNAs.

### 2.13 Statistical analysis

Statistical analysis was conducted using GraphPad Prism 8.0 and SPSS 26.0. Data are shown as mean ± standard deviation. Intergroup differences were assessed by t-test, one-way ANOVA, or chi square test. Statistically significant differences are shown as *P<0.05, **P<0.01, and ***P<0.001.

## 3 Results

### 3.1 circ-0001875 expression is downregulated in severe asthma

**Figure 1:**
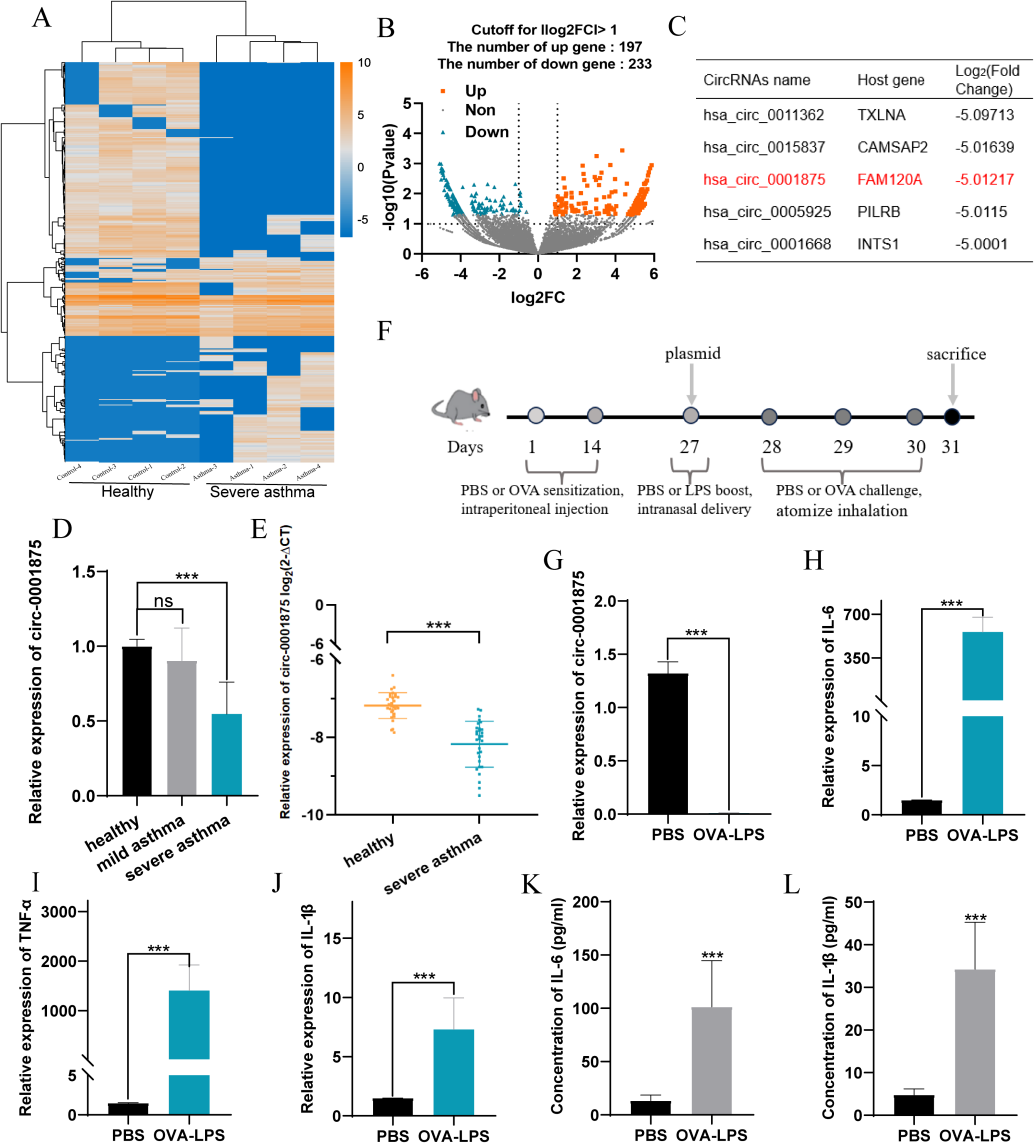
circ-0001875 expression in peripheral blood monocytes (PBMCs) from patients with asthma and an OVA-LPS-induced asthmatic mouse model. **(A)** Comparison of gene expression heatmaps between healthy and severe asthma groups. **(B)** Volcano plots showing differentially expressed circRNAs. **(C)** circRNAs with corresponding host genes and fold change values. **(D–E)** Differential expression of circ-0001875 in PBMCs from patients with mild asthma, patients with severe asthma, and healthy individuals. **(F)** OVA-LPS-induced mouse model of asthma. **(G)** circ-0001875 expression in lung tissue from the OVA-LPS-induced asthmatic mouse model. **(H–L)** Expression of M1 polarization–related inflammatory factors in lung tissue and BALF from the asthma model. The bars and error bars represent the mean ± SEM; ***P< 0.001. ns, not significant by unpaired Student's t-test or one-way ANOVA with Tukey's multiple comparisons test.

We conducted high-throughput sequencing of PBMCs from peripheral blood samples collected from patients with severe asthma and healthy individuals using (**Figure 1A**). Using the criteria of log2(fold-change) absolute value>1 and P-value<0.05, we identified 430 differentially expressed circRNAs, including 197 upregulated and 233 downregulated circRNAs (**Figure 1B**). The circRNAs differentially expressed between severe asthma and healthy control group are shown in **Supplementary Table S1**. Notably, as the linear counterpart of circ-0001875, FAM120A is associated with inflammation or asthma (48). circ-0001875 (has_circRNA_0001875) showed significant downregulation in the analyzed data. (**Figure 1C**).

We examined the expression level of circ-0001875 in PBMCs from patients with asthma and healthy individuals. The clinical characteristics of the subjects are shown in **Table 1**. We divided the patients with asthma into severe and mild asthma groups. Compared with patients with mild asthma, the patients with severe asthma showed a significant increase in inhaled steroid doses and decreased FENO (*P*<0.001), indicating a poorer response to inhaled corticosteroids. Lung function in the severe asthma group was also significantly lower than that seen in the mild asthma group (*P*<0.001). In addition, the sputum of patients with severe asthma patients contained more inflammatory cells than that of patients with mild asthma, including eosinophils, macrophages, lymphocytes, and neutrophils (*P*<0.001). Furthermore, circ-0001875 was downregulated in the PBMCs of patients with asthma, especially those with severe asthma (**Figures 1D, E**).

**Table 1:** Clinical characteristics of the study participants.

Variables	Severe asthma (n=34)	Mild-moderate Asthma (n=32)	Healthy (n=35)	*P* value
Age (y) mean (SD)	38.5 ± 14.7	43.7 ± 15.3	40.1 ± 10.2	0.292
Male n (%)	17 (50)	12 (37.5)	15 (42.9)	0.589
Disease duration years median (IQR)	13 (7,17.5)	5 (3,11.5)	NA	< 0.001
BMI kg/m2 mean (SD)	23.7 ± 2.6	23.6 ± 3.2	23.1 ± 2.4	0.631
Current smoking n (%)	8 (23.5)	6 (18.8)	7 (20)	0.883
ACT score, median (IQR)	13 (12,14)	22.5 (21.8,23)	NA	< 0.001
Beclometasone-equivalent dose of inhaled steroid (μg) median(IQR)	800 (800,800)	400 (400,500)	NA	< 0.001
Atopy* n (%)	26 (76.5)	26 (81.2)	1 (2.9)	< 0.001
Comorbidities Allergic rhinitis n (%)	25 (73.5)	22 (68.8)	1 (2.9)	< 0.001
Lung function:FEV1% predicted median(IQR)	71.4 (62,79.3)	97.2 (89.3,101.7)	106.4 (100.3,110)	< 0.001
FVC % predicted median(IQR)	93.5 (90,96.3)	105.6 (102.3,109.6)	112.3 (103.8,125.6)	< 0.001
FEV1/FVC % predicted Median (IQR)	79.8 (73.6,82)	86.2 (82.9,91.1)	84.7 (82.2,86.6)	< 0.001
FENO (ppb), median (IQR)	30.5 (21,46.8)	44.5 (27.2,57.2)	6 (4,7.5)	< 0.001
Total IgE (kUA/L), median (IQR)	897.5 (300.5,946.8)	467 (128,654.2)	41.2 (15.1,97.2)	< 0.001
Sputum total cell count x10^6^ ·mL−1 median (IQR)	5.4 (4.5,6.4)	5.4 (3.9,6)	6.2 (5.7,7.5)	< 0.001
Sputum neutrophils (%) median(IQR)	34.4 (25.8,35.9)	15.3 (13.7,16.4)	14.8 (14,18.3)	< 0.001
Sputum eosinophils (%) median(IQR)	6.2 (4.9,9.4)	3.1 (1.9,7.4)	0.2 (0.2,0.2)	< 0.001
Sputum macrophages (%) median(IQR)	66.9 (64.7,69.4)	65.2 (61.2,69.6)	80.5 (78.8,82.4)	< 0.001
Lymphocytes (%) median(IQR)	1 (0.9,1.1)	1.3 (1,1.7)	0.8 (0.6,1)	< 0.001
Epithelial cells (%) median(IQR)	4.5 (1.2)	4.7 (1.4)	3.4 (1.5)	< 0.001
WBC median (IQR)	5.9 (4.9,6.5)	5.7 (4.9,6.7)	3.6 (3,4.1)	< 0.001
EO# median (IQR)	0.4 (0.3,0.8)	0.3 (0.2,0.6)	0.2 (0.1,0.4)	0.002
neutrophils median (IQR)	5.3 (4.3,5.7)	3.6 (2.7,3.7)	3.5 (2.5,3.9)	< 0.001

The data are presented as the mean (standard deviation), median (interquartile range), or n (%). ACT, asthma control test; BMI, body mass index; DRSmethacholine, slope of the dose-response curve for methacholine provocation; FENO, fraction of exhaled nitric oxide; ICS, inhaled corticosteroids; IQR, Interquartile range; NA, not available; NS, not significant.

*Atopy is defined as a specific IgE level greater than 0.35 kU/L in response to inhaled allergens (Phadiatop).

Furthermore, we constructed an animal model of severe asthma by treating mice with LPS-OVA and found that circ-0001875 was expressed at low levels in these animals (**Figures 1F, G**) (46, 47). More importantly, inflammatory factors released by M1 macrophages were expressed at high levels in the lung tissue and BALF of the mouse model of severe asthma (**Figures 1H-L**).

### 3.2 Circ-0001875 inhibits M1 macrophage polarization in severe asthma

**Figure 2:**
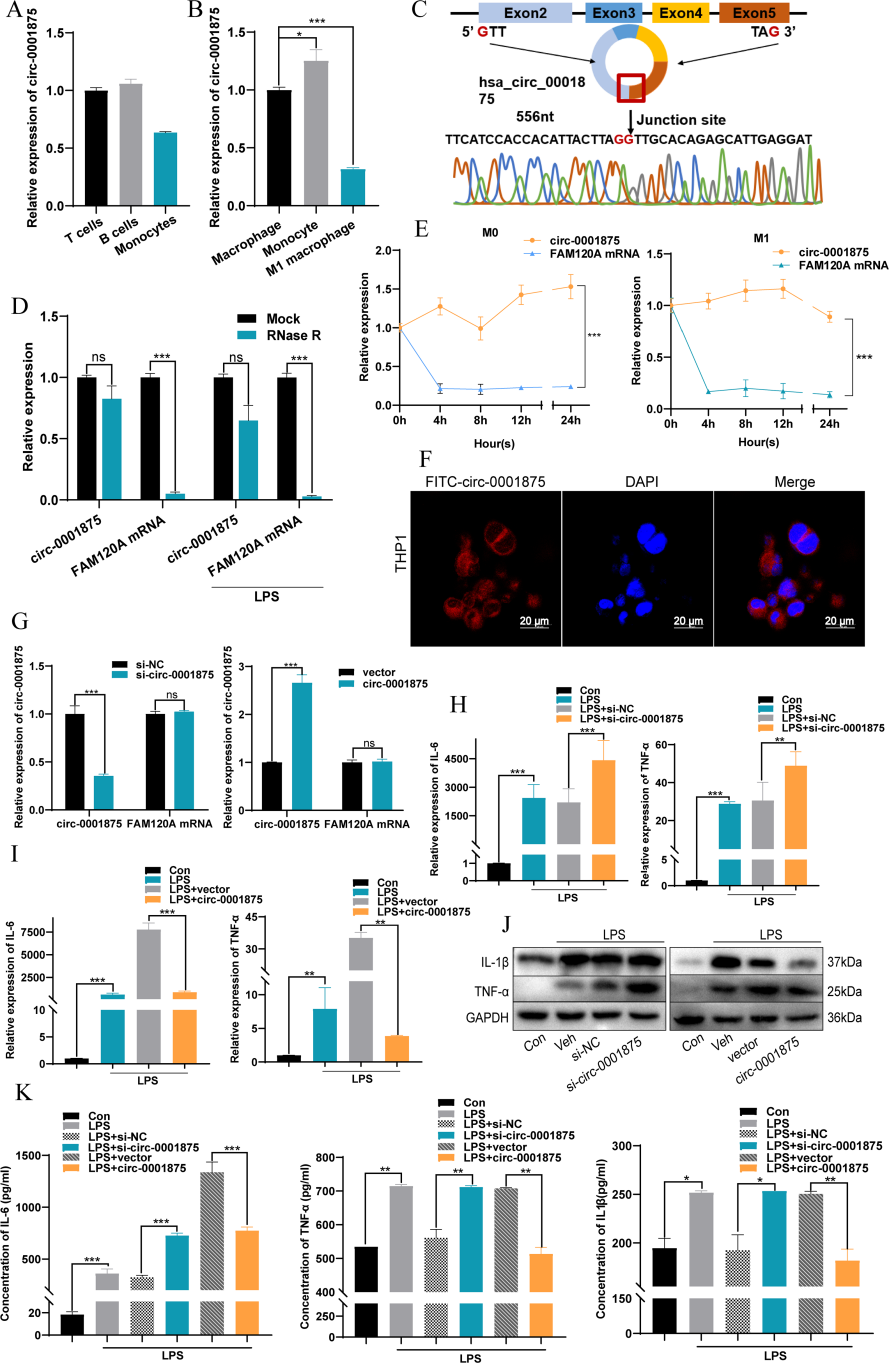
circ-0001875 inhibits M1 macrophage polarization *in vitro*. **(A–B)** circ-0001875 expression in different cells. **(C)** Schematic of the genomic location and back splicing of circ-0001875 with the splicing site validated by Sanger sequencing. **(D–E)** The expression of circ-0001875 and FAM120A mRNA after treatment with actinomycin D and RNase R. **(F)** Fluorescence microscopy images revealing cellular localization of FITC-circ_0001875. **(G)** circ-0001875 and FAM120A mRNA expression levels after circ-0001875 knockdown and overexpression. **(H–K)** The effect of circ-0001875 knockdown or overexpression on macrophage polarization was detected by RT-qPCR, Western blot, and ELISA, respectively. The bars and error bars represent the mean ± SEM; *P< 0.05, **P< 0.01, and ***P< 0.001. ns, not significant by one-way ANOVA with Tukey's multiple comparisons test.

Subsequently, we found that circ-0001875 was downregulated in monocytes isolated from peripheral blood and downregulated in human macrophages stimulated by LPS (**Figures 2A, B**). Monocytes are one source of macrophages in lung tissue, and macrophages are involved in severe asthma-induced pulmonary inflammation (11, 12, 49). Therefore, we hypothesized that circ-0001875 induces M1 macrophage polarization.

The sequence and structural composition of circ-0001875 were identified using the CircBase database (50), and divergent and convergent primers (circ-0001875 and FAM120A mRNA) were designed and synthesized to verify the stability of circ-0001875 as a circular RNA (**Figure 2C**). RNase R experiments and ActD experiments showed that circular circ-0001875 was more stable than linear FAM120A mRNA (**Figures 2D, E**). In addition, fluorescence *in situ* hybridization confirmed that circ-0001875 was mainly expressed in the cytoplasm, indicating its potential as a competitive endogenous RNA (**Figure 2F**).

To investigate the biological function of circ-0001875 in severe asthma, we designed and synthesized circ-0001875 siRNA and overexpression plasmids, which were then transfected into THP1 cells. The effectiveness of circ_0001875 knockdown and upregulation was validated in cells, showing no effect on the expression of its linear counterpart FAM120A (**Figure 2G**). *In vitro*, knocking down circ-0001875 promoted M1 macrophage polarization and the secretion of related inflammatory factors, while circ-0001875 overexpression inhibited M1 macrophage polarization (**Figures 2H-K**).

**Figure 3:**
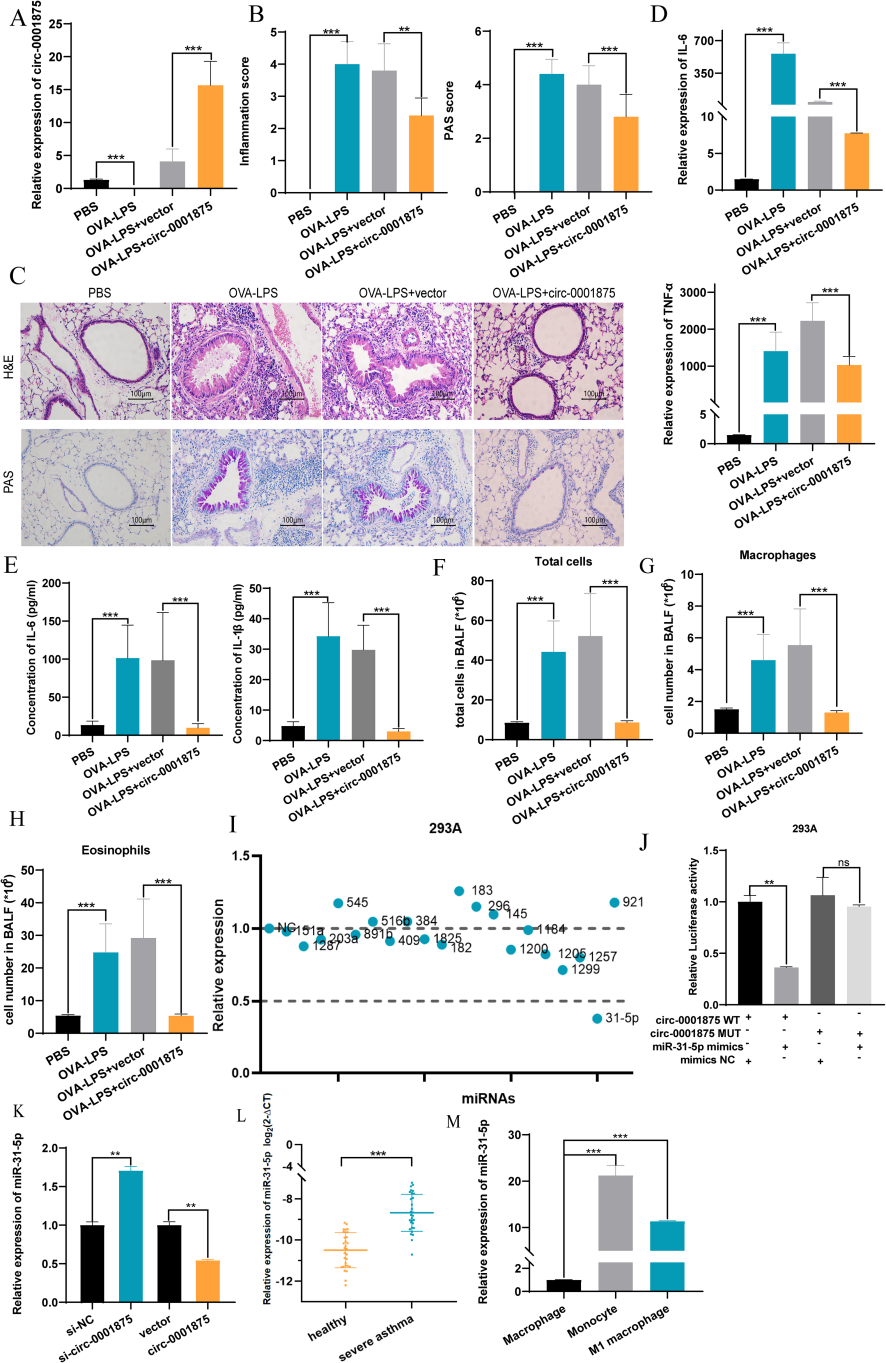
circ-0001875 overexpression inhibits pulmonary inflammation in an OVA-LPS-induced asthma model. **(A)** circ-0001875 expression in lung tissue from the mouse model. **(B, C)** Lung sections stained using hematoxylin and eosin (H&E) and periodic acid–Schiff (PAS). **(D, E)** The effect of circ-0001875 overexpression on macrophage polarization in the lung tissue. **(F-H)** Differential cell counts in BALF from the mouse model. **(I)** Relative expression of various miRNAs. **(J)** Relative luciferase activity of miR-31-5p mimics or miR-NC after co-transfection with pmirGLO-Wt-circ-0001875 or pmirGLO-MUT-circ-0001875 in 293A cells, respectively. **(K)** Differential expression of miR-31-5p after circ-0001875 knockdown and overexpression. **(L, M)** miR-31-5p expression in PBMCs, monocytes and macrophages. The bars and error bars represent the mean ± SEM; **P< 0.01, and ***P< 0.001. ns, not significant by unpaired Student's t-test or one-way ANOVA with Tukey's multiple comparisons test.

Next we constructed an animal model of severe asthma model by treating mice with OVA-LPS, while the negative control group was treated with PBS (46, 47). A CD68–circ-0001875 overexpression vector and negative control vector were transfected into the lungs of C57BL/6 mice via intrabronchial injection. Brief transfection with the CD68–circ-0001875 plasmid resulted in circ-0001875 overexpression in lung tissue (**Figure 3A**, **Supplementary Figure S1**). H&E and PAS staining confirmed an increase in inflammatory cell infiltration and goblet cell proliferation in the lung tissue of severely asthmatic mice, while circ-0001875 overexpression inhibited airway inflammation (**Figures 3B, C**). *In vivo*, circ-0001875 overexpression inhibited the expression of inflammatory factors related to M1 macrophage polarization (**Figures 3D, E**). More macrophages and eosinophils were present in BALF from the severe asthma group than BALF from the negative controls, while circ-0001875 overexpression reduced the number of macrophages and eosinophils in BALF (**Figures 3F-H**). These results indicate that circ-0001875 inhibits M1 macrophage polarization *in vivo*, thereby reducing airway inflammation in severe asthma.

### 3.3 circ-0001875 acts as a sponge for miR-31-5p, thereby reducing M1 macrophage polarization

Next we used CircBase (50) to search for potential miRNA targets of circ-0001875 and Circinteractome (51) to identify their interaction sites. We found 21 miRNA sequences with interaction regions that complemented circ-0001875 (**Figure 3I**). Dual luciferase reporter gene detection and rescue experiments showed that circ-0001875 can serve as a “molecular sponge” for miR-31-5p, exerting a negative regulatory effect on its expression (**Figures 3J, K**, **Supplementary Figure S2A**). miR-31–5 was expressed at higher levels in PBMCs of patients with severe asthma (**Figures 3L, M**).

**Figure 4:**
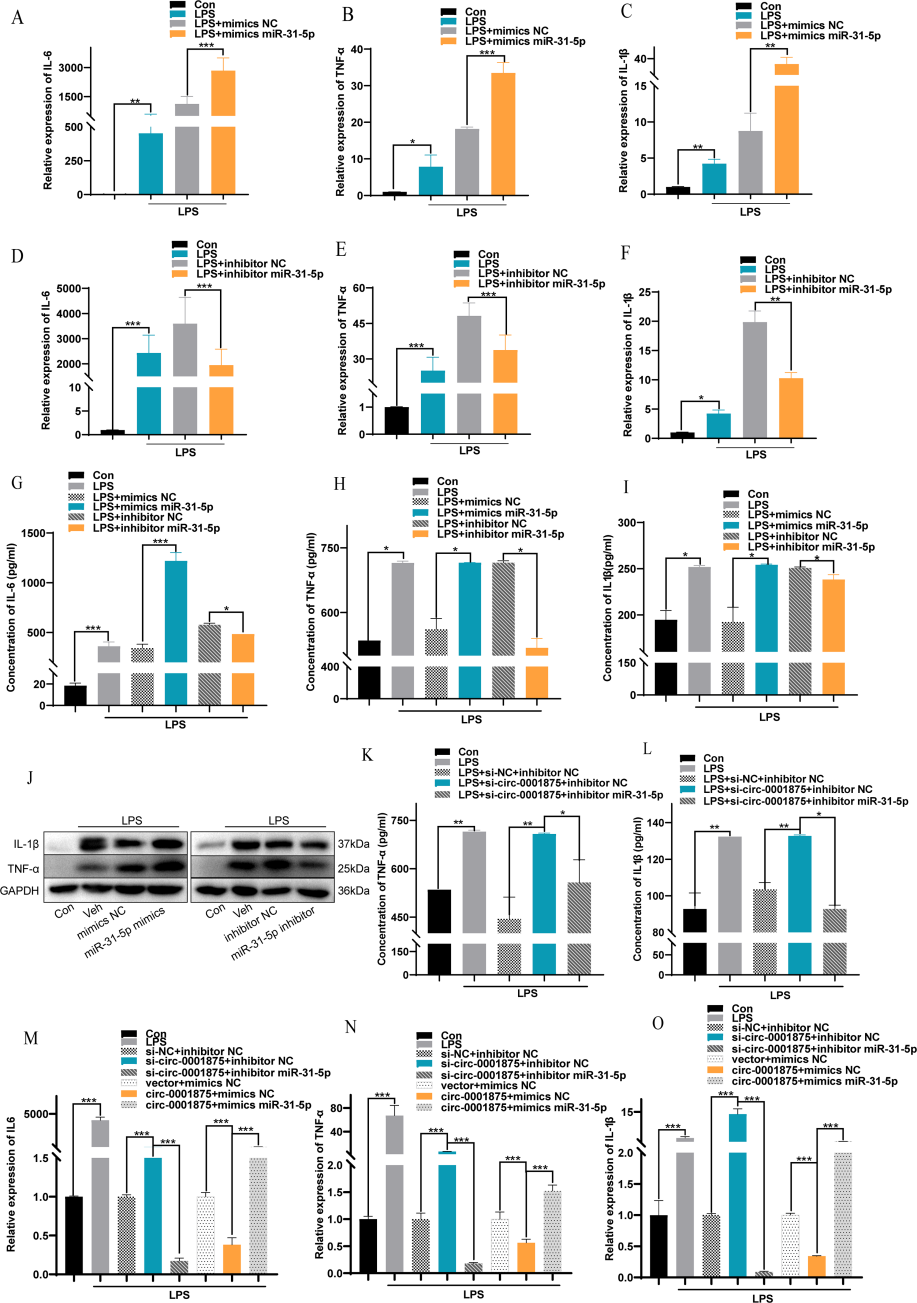
The interaction between circ-0001875 and miR-31-5p affects M1 macrophage polarization. **(A–F)** The effect of miR-31-5p mimics or an miR-31-5p inhibitor on the relative expression of IL-6, IL-1β and TNF-α. **(G–I)** The effect of miR-31-5p mimics and an miR-31-5p inhibitor on the concentration levels of IL-6, IL-1β and TNF-α. **(J)** The effects of miR-31-5p mimics and an miR-31-5p inhibitor on macrophage polarization were detected by Western blot. **(K–L)** ELISA was used to detect the effect of co-transfection with si-circ-0001875+miR-31-5p inhibitor and pc-circ-0001875+miR-31-5p mimics on macrophage polarization. **(M–O)** The effects of co-transfection with si-circ-0001875+miR-31-5p inhibitor and pc-circ-0001875+miR-31-5p mimics on macrophage polarization were detected by RT-qPCR. The bars and error bars represent the mean ± SEM; *P< 0.05, **P< 0.01, and ***P< 0.001.

To verify at the functional level that miR-31-5p is a downstream miRNA target of circ-0001875, we constructed miR-31-5p mimics and an miR-31-5p inhibitor to overexpress and inhibit miR-31-5p expression, respectively (**Supplementary Figure S2B**). miR-31-5p overexpression promoted M1 macrophage polarization, while inhibition of miR-31-5p expression inhibited M1 macrophage polarization (**Figures 4A-F**). In addition, miR-31-5p mimics promoted the secretion of M1 polarization–related inflammatory factors by macrophages, while miR-31-5p inhibition had the opposite effect (**Figures 4G-J**). Subsequent rescue experiments confirmed that miR-31-5p is a downstream miRNA of circ-0001875 involved in regulating macrophage M1 polarization (**Figures 4K-O**). These results indicate that circ-0001875 acts as a molecular sponge for miR-31-5p to regulate M1 macrophage polarization associated with severe asthma.

### 3.4 circ-0001875 affects M1 macrophage polarization by modulating SP1 expression

**Figure 5:**
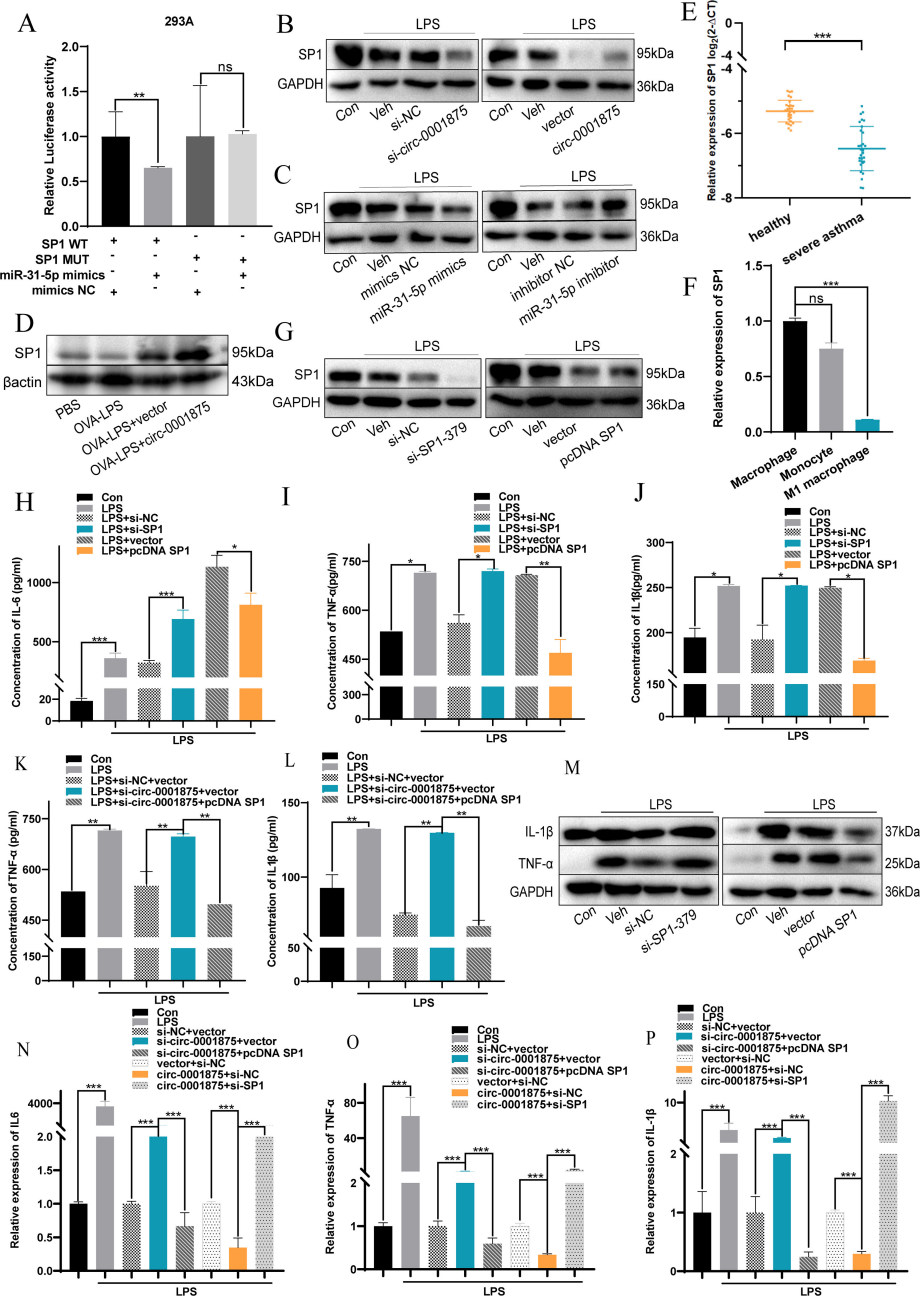
circ-0001875 acts as a sponge for miR-31-5p and targets SP1. **(A)** miR-31-5p mimics or miR-NC were co-transfected with pmirGLO-Wt-SP1 or pmirGLO-MUT-SP1, and relative luciferase activity was detected. **(B-C)** Expression of SP1 in THP1 cells transfected with circ-0001875 knockdown or overexpression plasmids and miR-31-5p mimics or an inhibitor. **(D)** SP1 expression in lung tissue. **(E)** SP1 expression in PBMCs from patients with severe asthma and healthy individuals. **(F)** SP1 expression in monocytes and M1-polarized macrophages. **(G)** The transfection efficiency of SP1 knockdown (si-SP1) or overexpression (pc-SP1). **(H-P)** The effects of si-SP1, pc-SP1, co-transfection with si-circ-0001875+pc-SP1 and pc-circ-0001875+si-SP1 on macrophage polarization. The bars and error bars represent the mean ± SEM; *P< 0.05, **P< 0.01, and ***P< 0.001. ns, not significant by unpaired Student's t-test or one-way ANOVA with Tukey's multiple comparisons test.

To understand the mechanisms underlying the observations described above, we used the online prediction databases (miRDB, TargetScan, and miRWalk (52–54) to predict potential downstream target genes of miR-31-5p. Intersection analysis identified potential complementary binding sites in 72 genes (**Supplementary Figure S3**). Subsequently, we searched the relevant literature and found that SP1 is a potential downstream target genes of miR-31-5p that may be related to M1 macrophage polarization. Next, based on the complementary pairing sequences between SP1 and miR-31-5p, we constructed wild-type (pmirGLO-Wt-SP1) and mutant (pmirGLO-MUT-SP1) dual luciferase reporting plasmids for SP1 and demonstrated that SP1 is downstream and negatively regulated by miR-31-5p (**Figures 5A-C**, **Supplementary Figures S4A, Supplementary Figures S5A, B**). Furthermore, SP1 was expressed at low levels in a mouse model of severe asthma, PBMCs from patients with severe asthma, and THP1 cells stimulated with LPS (**Figures 5D-F**).

To investigate the effects of SP1 on macrophage polarization, we constructed SP1 knockdown (si-SP1) and overexpression (pcDNA SP1) plasmids (**Figure 5G**, **Supplementary Figure S4B**). SP1 knockdown promoted M1 macrophage polarization, while SP1 overexpression had the opposite effect (**Supplementary Figures S4C–E**). Moreover, knocking down SP1 promoted the expression of M1-polarized cytokines by macrophages, including IL-6, IL-1β, and TNF-α, compared with the negative control group, while SP1 overexpression had the opposite effect (**Figures 5H-M**). A rescue experiment showed that si-SP1 reversed this inhibitory effect of circ-0001875 overexpression on M1 macrophage polarization. While knocking down circ-0001875 promoted M1 macrophage polarization, transfection with pcDNA SP1 inhibited this effect (**Figures 5N-P**). These results indicate that SP1 is located downstream of circ-0001875 and forms a regulatory axis with circ-0001875/miR-31-5p affecting M1 macrophage polarization in bronchial asthma.

### 3.5 Circ-0001875 regulates M1 macrophage polarization via the NF-κB signaling pathway

**Figure 6:**
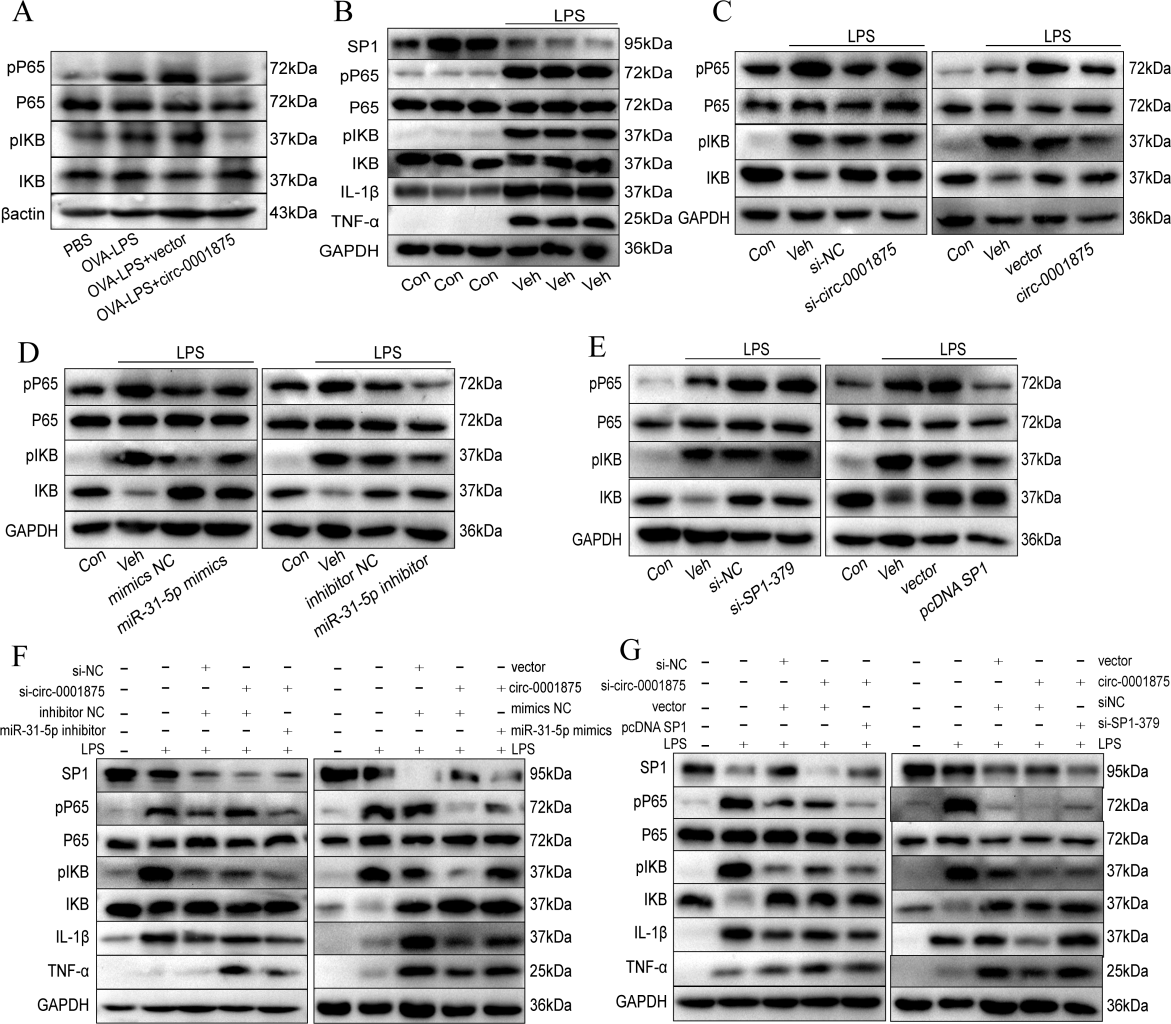
circ-0001875 alters M1 polarization via the NF-κB signaling pathway. **(A)** circ-0001875 alters M1 polarization via the NF-κB signaling pathway in a mouse model. **(B)** The expression levels of NF-κB signaling pathway and SP1 proteins in THP1 cells stimulated by LPS. **(C-E)** The effects of circ-0001875, miR-31-5p, and SP1 on the expression levels of NF-κB signaling pathway proteins. **(F-G)** The rescue experiment assessed the impact of circ-0001875, miR-31-5p, and SP1 on the expression levels of NF-κB signaling pathway and SP1 proteins.

To explore how the circ-0001875 regulatory axis affects M1 macrophage polarization, we performed a series of Western blot experiments. p-p65 and p-IKB were highly expressed in the lung tissue of the mouse model of severe asthma, while circ-0001875 overexpression inhibited their expression (**Figure 6A**). *In vitro*, we found that stimulating THP1 cells with LPS upregulated the expression of p-p65 and p-IKB, which are NF-κB signaling pathway proteins (**Figure 6B**). circ-0001875 knockdown further upregulated the expression of p-p65 and p-IKB, while circ-0001875 overexpression inhibited the expression of these protein (**Figure 6C**). SP1 and circ-0001875 had similar effects on the expression of NF-κB signaling pathway proteins, while miR-31-5p had an opposite effect (**Figures 6D, E**). Co-transfection of cells with circ-0001875 and miR-31-5p showed that miR-31-5p could restore the effects of circ-0001875 on p-p65 and p-IKB expression levels. Similarly, when circ-0001875 was co-transfected with SP1, SP1 reversed the effects of circ-0001875 on p-p65 and p-IKB expression levels (**Figures 6F, G**). These results indicate that NF-κB, a key signaling pathway involved in M1 macrophage polarization, mediates signaling by the circ-0001875/miR-31-5p/SP1 regulatory axis, which modulates M1 macrophage polarization in severe asthma.

## 4 Discussion

Corticosteroids, as first-line treatment for persistent asthma, can effectively control airway inflammation. However, patients with severe asthma frequently respond poorly to corticosteroid treatment (4–6). Research has shown that, although Th2-mediated eosinophil recruitment and airway hyperresponsiveness (AHR) can be inhibited by corticosteroid treatment, OVA- and LPS-induced, Th1-mediated AHR is resistant to corticosteroid treatment. Corticosteroid treatment completely suppresses Th2-driven inflammation, but only partially inhibits Th1-driven neutrophil recruitment. In our study, patients with severe asthma had a poorer response to glucocorticoid therapy than patients with mild asthma, and thus used higher doses of inhaled corticosteroids. Patients with severe asthma patients exhibited both poorer lung function and more severe airway inflammation. We found that circ-0001875 expression is related to asthma severity: circ-0001875 is expressed at low levels in the PBMCs of patients with asthma, especially severe asthma. Interestingly, previous studies have shown that FAM120A, as the its linear counterpart of circ-0001875, is associated with inflammation or asthma (48). We also searched for potential miRNA targets of circ-0001875. Among them, upregulation of miR-1287-5p inhibits LPS induced epithelial mesenchymal transition and secretion of pro-inflammatory cytokines in human nasal epithelial cells (55). MiR-1184 downregulation serves as a diagnostic biomarker for neonatal sepsis, regulating LPS induced inflammatory response by inhibiting IL-16 in monocytes (56, 57). It can also target TRADD to regulate inflammatory response and cell apoptosis (58). In addition, studies have shown that miR-31-5p is upregulated in children with asthma (59). Therefore, we further investigated the correlation between circ-0001875 and asthma. In patients with severe asthma, pro-inflammatory cytokines in the airway lumen and bronchial mucosa, including TNF-α, IL-1β, IL-6, and IL-8, are mainly produced by macrophages, while LPS can stimulate THP-1 cells to secrete pro-inflammatory cytokines (60–62). We found that circ-0001875 is expressed at low levels in a mouse model of severe asthma, and that M1 polarization–related inflammatory factors are expressed at high levels in the lung tissue and BALF of the same model.

In PBMCs isolated from peripheral blood, circ-0001875 was mainly downregulated in monocytes. Monocytes are a source of macrophages in lung tissue, and macrophages are involved in severe asthma-induced pulmonary inflammation (11, 12, 49). Therefore, we hypothesized that circ-0001875 regulates M1 macrophage polarization. During asthma progression, macrophages have various functions such as antigen presentation, cell clearance, production of inflammatory mediators, and pathogen clearance (7). Alveolar macrophages originate from fetal monocytes of embryonic origin. When damaged or depleted, monocytes are recruited from the circulation and differentiate into pulmonary macrophages (63–65). Emerging studies indicate that patients with asthma exhibit dysregulation of circRNAs, which regulate macrophage phenotype and function (19, 20, 66, 67). Here we found that circ-0001875 overexpression inhibited LPS-induced M1 macrophage polarization. Furthermore, circ-0001875 overexpression inhibited pulmonary inflammation in a severe asthma model. We found that circ-0001875 overexpression inhibited macrophage secretion of pro-inflammatory cytokines *in vitro* and *in vivo*.

CircRNAs can act as molecular sponges to regulate biological processes such as macrophage activation and Th1/Th2 immune balance, thereby affecting airway inflammation (67, 68). For example, Shang et al. found that circ-0001359 was significantly downregulated in OVA-treated mice, and also acted as a specific sponge for miR-183-5p, thereby promotingFoxO1 expression and reducing airway remodeling by decreasing the secretion of inflammatory cytokines induced by M1 macrophage activation and pulmonary fibrosis (69). Similarly, a study showed that circ-0001326 promotes M1 macrophage polarization by directly regulating the miR-136-5p/USP4 axis, thereby promoting the secretion of inflammatory cytokines (70). In this study, circ-0001875 was mainly expressed in the cytoplasm, indicating its potential for sponge-like activity toward miRNAs in macrophages. There is evidence to suggest that miR-31-5p is highly expressed in the lung tissues of asthmatic mice and children (59). We also observed high levels of miR-31-5p expression in the PBMCs of asthma patients. We confirmed by luciferase assay that circ-0001875 acts a molecular sponge for miR-31-5p and can negatively regulate its expression. Furthermore, we found that miR-31-5p mimetics promote M1 macrophage polarization. Our findings indicate that miR-31-5p inhibitors counteract the promotion of M1 polarization caused by circ-0001875 knockdown, while circ-0001875 overexpression inhibits M1 polarization, and this effect is reversed by miR-31-5p overexpression. Our results indicate that circ-0001875, an miR-31-5p sponge, is crucial for M1 macrophage polarization–related airway inflammation.

SP1 can be induced and activated by LPS in the human monocyte line THP-1 (34, 35). Although previous studies have reported that SP1 typically promotes pro-inflammatory responses, there are also studies indicating that SP1 has anti-inflammatory effects (71, 72). Multiple studies have shown that SP1, as a macrophage transcription factor, is involved in macrophage activation and inflammation-related cytokine release (73–75). Costa et al. found that miR-31-5p targets SP1 in osteoblasts and chondrocytes, promoting the release of inflammatory cytokines in joints (76). In our study, we found that SP1 is significantly downregulated in M1 macrophages and PBMCs from patients with asthma, and its expression pattern is similar to that of circ-0001875. We then asked whether the sponge-like activity of circ-0001875 toward miR-31-5p inhibits macrophage M1 polarization by targeting SP1 and found that SP1 is a downstream target gene of circ-0001875/miR-31-5p. Based on our finding that SP1 expression negatively regulates M1 polarization, we further investigated the effect of circ-0001875 on SP1-activated macrophages. We found that SP1 overexpression restored the M1 macrophage polarization induced by knocking down circ-0001875, while knocking down SP1 had the opposite effect. In summary, circ-0001875 serves as a sponge for miR-31-5p, whose downstream target gene is SP1, which regulates M1 macrophage polarization, thereby affecting airway inflammation caused by macrophage activation.

We previously reported that the NF-κB pathway is a major pathway involved in M1 macrophage polarization and helps regulate asthma-related airway inflammation and remodeling (13). SP1 is present in the enhancers or promoters of the HIV, ICAM-1, and GM-CSF genes, which regulate the NF-κB signaling pathway (35–39). In addition, SP1 can bind to NF-κB (42). Studies have shown that SP1 protects cardiomyocytes from inflammatory damage in atherosclerosis by inhibiting the NF-κB signaling pathway (77). In this study, OVA-LPS activated the NF-κB signaling pathway in a severe asthma model, and knocking down SP1 further upregulated the expression of NF-κB signaling pathway proteins. circ-0001875 had a similar effect on the expression levels of NF-κB signaling pathway proteins as SP1, while miR-31-5p had the opposite effect. Similar results were obtained in our *in vitro* experiments. These results suggest that the circ-0001875/miR-31-5p/SP1 regulatory axis influences M1 macrophage polarization of associated with bronchial asthma through the NF-κB signaling pathway. Corticosteroids are effective inhibitors of NF-κB activity, and glucocorticoid receptors directly bind to NF-κB to inhibit NF-κB-mediated gene activation (78, 79). Therefore, NF-κB activity reflects the effectiveness of glucocorticoid therapy. In our study, circ-0001875 expression was inversely associated with asthma severity. We also demonstrated that circ-0001875 can inhibit NF-κB activity. circ-0001875 may therefore act synergistically with glucocorticoids to control Th1-induced airway inflammation.

Our research still has some limitations.The inter individual heterogeneity of PBMC samples (such as age, immune status, etc.) may further increase variability. Although we reduce confounding factors through strict matching criteria such as age and gender, small sample sizes may still limit the comprehensive evaluation of population level circRNA expression patterns. Previous studies have indicated that luciferase, rescue, and expression data can demonstrate axis relevance (80, 81). However, RIP assays or CRISPR/Cas13 knockdown can further enhance the correlation (82).

## 5 Conclusion

Our findings indicate that circ-0001875 is downregulated in the PBMCs of patients with severe asthma and is associated with asthma severity. Mechanistically, circ-0001875 acts as a sponge for miR-31-5p, co-targeting SP1 and participating in M1 macrophage polarization through the NF-κB signaling pathway, thereby affecting airway inflammation. circ-0001875 downregulation in severe asthma highlights the fact that dysregulated circRNAs mediate asthma pathophysiology by regulating M1 macrophage polarization associated with severe pro-inflammatory response. This provides new experimental evidence for understanding the role of circRNA in asthma. Subsequent research needs to validate its translational medicine potential through larger clinical samples and preclinical models.

## Data Availability

The raw data supporting the conclusions of this article will be made available by the authors, without undue reservation.

## References

[1] Jeffery PK. Remodeling in asthma and chronic obstructive lung disease. Am J Respir Crit Care Med. (2001) 164:S28–38. doi: 10.1164/ajrccm.164.supplement_2.2106061 11734464

[2] Lambrecht BN Hammad H Fahy JV. The cytokines of asthma. Immunity. (2019) 50:975–91. doi: 10.1016/j.immuni.2019.03.018 30995510

[3] Masoli M Fabian D Holt S Beasley R. Global Initiative for Asthma (GINA) Program. The global burden of asthma: executive summary of the GINA Dissemination Committee report. Allergy. (2004) 59:469–78. doi: 10.1111/j.1398-9995.2004.00526.x 15080825

[4] National Asthma Education and Prevention Program. National asthma education and prevention program. Expert panel report: guidelines for the diagnosis and management of asthma update on selected topics–2002. J Allergy Clin Immunol. (2002) 110:S141–219. 12542074

[5] Ito K Chung KF Adcock IM. Update on glucocorticoid action and resistance. J Allergy Clin Immunol. (2006) 117:522–43. doi: 10.1016/j.jaci.2006.01.032 16522450

[6] Sher ER Leung DY Surs W Kam JC Zieg G Kamada AK. Steroid-resistant asthma. Cellular mechanisms contributing to inadequate response to glucocorticoid therapy. J Clin Invest. (1994) 93:33–9. doi: 10.1172/JCI116963 PMC293719 8282803

[7] van der Veen TA de Groot LES Melgert BN. The different faces of the macrophage in asthma. Curr Opin Pulm Med. (2020) 26:62–8. doi: 10.1097/MCP.0000000000000647 PMC6903353 31703000

[8] Li H Ciric B Yang J Xu H Fitzgerald DC Elbehi M. Intravenous tolerance modulates macrophage classical activation and antigen presentation in experimental autoimmune encephalomyelitis. J Neuroimmunol. (2009) 208:54–60. doi: 10.1016/j.jneuroim.2009.01.002 19187972 PMC2723950

[9] Liu C Li Y Yu J Feng L Hou S Liu Y. Targeting the shift from M1 to M2 macrophages in experimental autoimmune encephalomyelitis mice treated with fasudil. PloS One. (2013) 8:e54841. doi: 10.1371/journal.pone.0054841 23418431 PMC3572131

[10] Sica A Mantovani A. Macrophage plasticity and polarization: *in vivo* veritas. J Clin Invest. (2012) 122:787–95. doi: 10.1172/JCI59643 PMC3287223 22378047

[11] Kim Y-K Oh S-Y Jeon SG Park H-W Lee S-Y Chun E-Y. Airway exposure levels of lipopolysaccharide determine type 1 versus type 2 experimental asthma. J Immunol. (2007) 178:5375–82. doi: 10.4049/jimmunol.178.8.5375 17404323

[12] Naura AS Zerfaoui M Kim H Abd Elmageed ZY Rodriguez PC Hans CP. Requirement for inducible nitric oxide synthase in chronic allergen exposure-induced pulmonary fibrosis but not inflammation. J Immunol. (2010) 185:3076–85. doi: 10.4049/jimmunol.0904214 PMC3077076 20668217

[13] Huang D Sun C Chen M Bai S Zhao X Wang W. Bergenin ameliorates airway inflammation and remodeling in asthma by activating SIRT1 in macrophages to regulate the NF-κB pathway. Front Pharmacol. (2022) 13:994878. doi: 10.3389/fphar.2022.994878 36313381 PMC9606584

[14] Liang Q Fu J Wang X Liu L Xiao W Gao Y. circS100A11 enhances M2a macrophage activation and lung inflammation in children with asthma. Allergy. (2023) 78:1459–72. doi: 10.1111/all.15515 36104951

[15] Yang Y Huang G Xu Q Zhao G Jiang J Li Y. miR-146a-5p attenuates allergic airway inflammation by inhibiting the NLRP3 inflammasome activation in macrophages. Int Arch Allergy Immunol. (2022) 183:919–30. doi: 10.1159/000524718 35660690

[16] Zhu X He L Li X Pei W Yang H Zhong M. LncRNA AK089514/miR-125b-5p/TRAF6 axis mediates macrophage polarization in allergic asthma. BMC Pulm Med. (2023) 23:45. doi: 10.1186/s12890-023-02339-1 36717790 PMC9887860

[17] Liu X Ali MK Dua K Mao Y Liu J. Circular RNAs: emerging players in asthma and COPD. Front Cell Dev Biol. (2023) 11:1267792. doi: 10.3389/fcell.2023.1267792 38078005 PMC10704470

[18] Tian C Gao J Yang L Yuan X. Non-coding RNA regulation of macrophage function in asthma. Cell Signal. (2023) 112:110926. doi: 10.1016/j.cellsig.2023.110926 37848099

[19] Sufianov A Bessonova M Begliarzade S Kudriashov V Danilov A Ilyasova T. Studies on the role of non-coding RNAs in controlling the activity of T cells in asthma. Non-coding RNA Res. (2023) 8:211–7. doi: 10.1016/j.ncrna.2023.02.004 PMC9972402 36865391

[20] Xiao B Li L Yao D Mo B. Noncoding RNAs in asthmatic airway smooth muscle cells. Eur Respir Rev. (2023) 32:220184. doi: 10.1183/16000617.0184-2022 37076176 PMC10113956

[21] Kristensen LS Andersen MS Stagsted LVW Ebbesen KK Hansen TB Kjems J. The biogenesis, biology and characterization of circular RNAs. Nat Rev Genet. (2019) 20:675–91. doi: 10.1038/s41576-019-0158-7 31395983

[22] Hansen TB Jensen TI Clausen BH Bramsen JB Finsen B Damgaard CK. Natural RNA circles function as efficient microRNA sponges. Nature. (2013) 495:384–8. doi: 10.1038/nature11993 23446346

[23] Das A Ganesh K Khanna S Sen CK Roy S. Engulfment of apoptotic cells by macrophages: a role of microRNA-21 in the resolution of wound inflammation. J Immunol. (2014) 192:1120–9. doi: 10.4049/jimmunol.1300613 PMC4358325 24391209

[24] Bazzoni F Rossato M Fabbri M Gaudiosi D Mirolo M Mori L. Induction and regulatory function of miR-9 in human monocytes and neutrophils exposed to proinflammatory signals. Proc Natl Acad Sci U.S.A. (2009) 106:5282–7. doi: 10.1073/pnas.0810909106 PMC2664036 19289835

[25] Tx L AM Me R. MicroRNA-21 is up-regulated in allergic airway inflammation and regulates IL-12p35 expression. J Immunol (Baltimore Md : 1950). (2009) 182(8):4994–5002. doi: 10.4049/jimmunol.0803560 PMC4280862 19342679

[26] Lawrence T Natoli G. Transcriptional regulation of macrophage polarization: enabling diversity with identity. Nat Rev Immunol. (2011) 11:750–61. doi: 10.1038/nri3088 22025054

[27] Martinez-Nunez RT Louafi F Sanchez-Elsner T. The interleukin 13 (IL-13) pathway in human macrophages is modulated by microRNA-155 via direct targeting of interleukin 13 receptor alpha1 (IL13Ralpha1). J Biol Chem. (2011) 286:1786–94. doi: 10.1074/jbc.M110.169367 PMC3023473 21097505

[28] Yoon WH Meinhardt H Montell DJ. miRNA-mediated feedback inhibition of JAK/STAT morphogen signalling establishes a cell fate threshold. Nat Cell Biol. (2011) 13:1062–9. doi: 10.1038/ncb2316 PMC3167036 21857668

[29] Graff JW Dickson AM Clay G McCaffrey AP Wilson ME. Identifying functional microRNAs in macrophages with polarized phenotypes. J Biol Chem. (2012) 287:21816–25. doi: 10.1074/jbc.M111.327031 PMC3381144 22549785

[30] Maneechotesuwan K. Role of microRNA in severe asthma. Respir Invest. (2019) 57:9–19. doi: 10.1016/j.resinv.2018.10.005 30455067

[31] Gil-Martínez M Lorente-Sorolla C Naharro S Rodrigo-Muñoz JM Del Pozo V. Advances and highlights of miRNAs in asthma: biomarkers for diagnosis and treatment. Int J Mol Sci. (2023) 24:1628. doi: 10.3390/ijms24021628 36675145 PMC9862966

[32] Cook T Gebelein B Urrutia R. Sp1 and its likes: biochemical and functional predictions for a growing family of zinc finger transcription factors. Ann N Y Acad Sci. (1999) 880:94–102. doi: 10.1111/j.1749-6632.1999.tb09513.x 10415854

[33] Black AR Black JD Azizkhan-Clifford J. Sp1 and krüppel-like factor family of transcription factors in cell growth regulation and cancer. J Cell Physiol. (2001) 188:143–60. doi: 10.1002/jcp.1111 11424081

[34] Chanteux H Guisset AC Pilette C Sibille Y. LPS induces IL-10 production by human alveolar macrophages via MAPKinases- and Sp1-dependent mechanisms. Respir Res. (2007) 8:71. doi: 10.1186/1465-9921-8-71 17916230 PMC2080632

[35] Ma W Lim W Gee K Aucoin S Nandan D Kozlowski M. The p38 mitogen-activated kinase pathway regulates the human interleukin-10 promoter via the activation of Sp1 transcription factor in lipopolysaccharide-stimulated human macrophages. J Biol Chem. (2001) 276:13664–74. doi: 10.1074/jbc.M011157200 11278848

[36] Perkins ND Edwards NL Duckett CS Agranoff AB Schmid RM Nabel GJ. A cooperative interaction between NF-kappa B and Sp1 is required for HIV-1 enhancer activation. EMBO J. (1993) 12:3551–8. doi: 10.1002/j.1460-2075.1993.tb06029.x PMC413631 8253080

[37] Johnson JP Stade BG Hupke U Holzmann B Riethmüller G. The melanoma progression-associated antigen P3.58 is identical to the intercellular adhesion molecule, ICAM-1. Immunobiology. (1988) 178:275–84. doi: 10.1016/S0171-2985(88)80071-8 3229781

[38] Neish AS Williams AJ Palmer HJ Whitley MZ Collins T. Functional analysis of the human vascular cell adhesion molecule 1 promoter. J Exp Med. (1992) 176:1583–93. doi: 10.1084/jem.176.6.1583 PMC2119448 1281211

[39] Jones KA Kadonaga JT Luciw PA Tjian R. Activation of the AIDS retrovirus promoter by the cellular transcription factor, Sp1. Science. (1986) 232:755–9. doi: 10.1126/science.3008338 3008338

[40] Thompson LJ Dunstan SJ Dolecek C Perkins T House D Dougan G. Transcriptional response in the peripheral blood of patients infected with Salmonella enterica serovar Typhi. Proc Natl Acad Sci U.S.A. (2009) 106:22433–8. doi: 10.1073/pnas.0912386106 PMC2792164 20018727

[41] Kirbis S Breskvar UD Sabovic M Zupan I Sinkovic A. Inflammation markers in patients with coronary artery disease–comparison of intracoronary and systemic levels. Wien Klin Wochenschr. (2010) 122 Suppl 2:31–4. doi: 10.1007/s00508-010-1343-z 20517668

[42] Wang T Lafuse WP Zwilling BS. NFkappaB and Sp1 elements are necessary for maximal transcription of toll-like receptor 2 induced by Mycobacterium avium. J Immunol. (2001) 167:6924–32. doi: 10.4049/jimmunol.167.12.6924 11739511

[43] Yang L Sun K Chu J Qu Y Zhao X Yin H. Long non-coding RNA FTH1P3 regulated metastasis and invasion of esophageal squamous cell carcinoma through SP1/NF-kB pathway. BioMed Pharmacother. (2018) 106:1570–7. doi: 10.1016/j.biopha.2018.07.129 30119232

[44] Wu D Chen T Zhao X Huang D Huang J Huang Y. HIF1α-SP1 interaction disrupts the circ-0001875/miR-31-5p/SP1 regulatory loop under a hypoxic microenvironment and promotes non-small cell lung cancer progression. J Exp Clin Cancer Res. (2022) 41:156. doi: 10.1186/s13046-022-02336-y 35473752 PMC9044860

[45] (2020) 43:1023–48. doi: 10.3760/cma.j.cn112147-20200618-00721.

[46] Mao Z Qian Y Liu Z Shi Y Fan L Zhang Q. LINC00158 modulates the function of BEAS-2B cells via targeting BCL11B and ameliorates OVA-LPS-induced severe asthma in mice models. Int Immunopharmacol. (2024) 130:111739. doi: 10.1016/j.intimp.2024.111739 38442574

[47] Chen Y-R Xiang X-D Sun F Xiao B-W Yan M-Y Peng B. Simvastatin reduces NETosis to attenuate severe asthma by inhibiting PAD4 expression. Oxid Med Cell Longev. (2023) 2023:1493684. doi: 10.1155/2023/1493684 36778209 PMC9911252

[48] Ma J Meng Q Zhan J Wang H Fan W Wang Y. Paeoniflorin suppresses rheumatoid arthritis development via modulating the circ-FAM120A/miR-671-5p/MDM4 axis. Inflammation. (2021) 44:2309–22. doi: 10.1007/s10753-021-01504-0 34423389

[49] Hashimoto D Chow A Noizat C Teo P Beasley MB Leboeuf M. Tissue-resident macrophages self-maintain locally throughout adult life with minimal contribution from circulating monocytes. Immunity. (2013) 38:792–804. doi: 10.1016/j.immuni.2013.04.004 23601688 PMC3853406

[50] circBase . Available online at: http://circbase.org/ (Accessed March 7, 2024).

[51] circintercome . Available online at: https://circinteractome.nia.nih.gov/ (Accessed March 7, 2024).

[52] miRBase . Available online at: https://mirbase.org/ (Accessed March 7, 2024).

[53] TargetScanHuman 8.0 . Available online at: https://www.targetscan.org/vert_80/ (Accessed March 7, 2024).

[54] Liu W Wang X. Prediction of functional microRNA targets by integrative modeling of microRNA binding and target expression data. Genome Biol. (2019) 20:18. doi: 10.1186/s13059-019-1629-z 30670076 PMC6341724

[55] Hao W Zhu Y Guo Y Wang H. miR-1287-5p upregulation inhibits the EMT and pro-inflammatory cytokines in LPS-induced human nasal epithelial cells (HNECs). Transpl Immunol. (2021) 68:101429. doi: 10.1016/j.trim.2021.101429 34139308

[56] Xie W Wang Z Guo X Guan H. MiR-409-3p regulates the proliferation and apoptosis of THP-1 through targeting Rab10. Leuk Res. (2023) 132:107350. doi: 10.1016/j.leukres.2023.107350 37437422

[57] Wang D Han L. Downregulation of miR-1184 serves as a diagnostic biomarker in neonatal sepsis and regulates LPS-induced inflammatory response by inhibiting IL-16 in monocytes. Exp Ther Med. (2021) 21:350. doi: 10.3892/etm.2021.9781 33732323 PMC7903473

[58] Ling P Tang R Wang H Deng X Chen J. miR-1184 regulates inflammatory responses and cell apoptosis by targeting TRADD in an LPS-induced cell model of sepsis. Exp Ther Med. (2021) 21:630. doi: 10.3892/etm.2021.10062 33936286 PMC8082660

[59] Shi Z-G Sun Y Wang K-S Jia J-D Yang J Li Y-N. Effects of miR-26a/miR-146a/miR-31 on airway inflammation of asthma mice and asthma children. Eur Rev Med Pharmacol Sci. (2019) 23:5432–40. doi: 10.26355/eurrev_201906_18212 31298396

[60] Chanput W Mes J Vreeburg RAM Savelkoul HFJ Wichers HJ. Transcription profiles of LPS-stimulated THP-1 monocytes and macrophages: a tool to study inflammation modulating effects of food-derived compounds. Food Funct. (2010) 1:254–61. doi: 10.1039/c0fo00113a 21776474

[61] Hoshi H Ohno I Honma M Tanno Y Yamauchi K Tamura G. IL-5, IL-8 and GM-CSF immunostaining of sputum cells in bronchial asthma and chronic bronchitis. Clin Exp Allergy. (1995) 25:720–8. doi: 10.1111/j.1365-2222.1995.tb00009.x 7584683

[62] Ackerman V Marini M Vittori E Bellini A Vassali G Mattoli S. Detection of cytokines and their cell sources in bronchial biopsy specimens from asthmatic patients. Relationship to atopic status, symptoms, and level of airway hyperresponsiveness. Chest. (1994) 105:687–96. doi: 10.1378/chest.105.3.687 8131526

[63] Okuma T Terasaki Y Kaikita K Kobayashi H Kuziel WA Kawasuji M. C-C chemokine receptor 2 (CCR2) deficiency improves bleomycin-induced pulmonary fibrosis by attenuation of both macrophage infiltration and production of macrophage-derived matrix metalloproteinases. J Pathol. (2004) 204:594–604. doi: 10.1002/path.1667 15538737

[64] Tsou C-L Peters W Si Y Slaymaker S Aslanian AM Weisberg SP. Critical roles for CCR2 and MCP-3 in monocyte mobilization from bone marrow and recruitment to inflammatory sites. J Clin Invest. (2007) 117:902–9. doi: 10.1172/JCI29919 PMC1810572 17364026

[65] Guilliams M De Kleer I Henri S Post S Vanhoutte L De Prijck S. Alveolar macrophages develop from fetal monocytes that differentiate into long-lived cells in the first week of life via GM-CSF. J Exp Med. (2013) 210:1977–92. doi: 10.1084/jem.20131199 PMC3782041 24043763

[66] Ghafouri-Fard S Shoorei H Taheri M Sanak M. Emerging role of non-coding RNAs in allergic disorders. Biomedicine Pharmacotherapy. (2020) 130:110615. doi: 10.1016/j.biopha.2020.110615 32777705

[67] Liang J Liu X-H Chen X-M Song X-L Li W Huang Y. Emerging roles of non-coding RNAs in childhood asthma. Front Pharmacol. (2022) 13:856104. doi: 10.3389/fphar.2022.856104 35656293 PMC9152219

[68] Bao H Zhou Q Li Q Niu M Chen S Yang P. Differentially expressed circular RNAs in a murine asthma model. Mol Med Rep. (2020) 22:5412–22. doi: 10.3892/mmr.2020.11617 PMC7647044 33173985

[69] Shang Y Sun Y Xu J Ge X Hu Z Xiao J. Exosomes from mmu_circ_0001359-modified ADSCs attenuate airway remodeling by enhancing foxO1 signaling-mediated M2-like macrophage activation. Mol Ther - Nucleic Acids. (2020) 19:951–60. doi: 10.1016/j.omtn.2019.10.049 PMC6997502 32018116

[70] Gong B Zheng Y Li J Lei H Liu K Tang J. Luteolin activates M2 macrophages and suppresses M1 macrophages by upregulation of hsa_circ_0001326 in THP-1 derived macrophages. Bioengineered. (2022) 13:5079–90. doi: 10.1080/21655979.2022.2036897 PMC8973855 35152837

[71] Xu L Hu W Zhang J Qu J. Knockdown of versican 1 in lung fibroblasts aggravates Lipopolysaccharide-induced acute inflammation through up-regulation of the SP1-Toll-like Receptor 2-NF-κB Axis: a potential barrier to promising Versican-targeted therapy. Int Immunopharmacol. (2023) 121:110406. doi: 10.1016/j.intimp.2023.110406 37311354

[72] Liu Z-M Wang X Li C-X Liu X-Y Guo X-J Li Y. SP1 promotes HDAC4 expression and inhibits HMGB1 expression to reduce intestinal barrier dysfunction, oxidative stress, and inflammatory response after sepsis. J Innate Immun. (2022) 14:366–79. doi: 10.1159/000518277 PMC9274949 35780770

[73] Brightbill HD Plevy SE Modlin RL Smale ST. A prominent role for Sp1 during lipopolysaccharide-mediated induction of the IL-10 promoter in macrophages. J Immunol. (2000) 164:1940–51. doi: 10.4049/jimmunol.164.4.1940 10657644

[74] Feng P Che Y Gao C Chu X Li Z Li L. Profibrotic role of transcription factor SP1 in cross-talk between fibroblasts and M2 macrophages. iScience. (2023) 26:108484. doi: 10.1016/j.isci.2023.108484 38094246 PMC10716550

[75] Zheng X Sarode P Weigert A Turkowski K Chelladurai P Günther S. The HDAC2-SP1 axis orchestrates protumor macrophage polarization. Cancer Res. (2023) 83:2345–57. doi: 10.1158/0008-5472.CAN-22-1270 37205635

[76] Costa V De Fine M Carina V Conigliaro A Raimondi L De Luca A. How miR-31-5p and miR-33a-5p Regulates SP1/CX43 Expression in Osteoarthritis Disease: Preliminary Insights. Int J Mol Sci. (2021) 22:2471. doi: 10.3390/ijms22052471 33671114 PMC7957523

[77] Zhu Z Zhang G Li D Yin X Wang T. Silencing of specificity protein 1 protects H9c2 cells against lipopolysaccharide-induced injury via binding to the promoter of chemokine CXC receptor 4 and suppressing NF-κB signaling. Bioengineered. (2022) 13:3395–409. doi: 10.1080/21655979.2022.2026548 PMC8973921 35048778

[78] Hudson WH de VIMS JC N ER W AG H Yang Q. Cryptic glucocorticoid receptor-binding sites pervade genomic NF-κB response elements. Nat Commun. (2018) 9:1337. doi: 10.1038/s41467-018-03780-1 29626214 PMC5889392

[79] Ray A Prefontaine KE. Physical association and functional antagonism between the p65 subunit of transcription factor NF-kappa B and the glucocorticoid receptor. Proc Natl Acad Sci U.S.A. (1994) 91:752–6. doi: 10.1073/pnas.91.2.752 PMC43027 8290595

[80] Qi S Li F Yang L Liu P Guo L. Circ_0000215 aggravates cerebral ischemic vertigo by targeting miR-361-3p to promote neuroinflammation and apoptosis. J Stroke Cerebrovasc Dis. (2025) 34:108317. doi: 10.1016/j.jstrokecerebrovasdis.2025.108317 40239828

[81] Jia Y Li X Nan A Zhang N Chen L Zhou H. Circular RNA 406961 interacts with ILF2 to regulate PM2.5-induced inflammatory responses in human bronchial epithelial cells via activation of STAT3/JNK pathways. Environ Int. (2020) 141:105755. doi: 10.1016/j.envint.2020.105755 32388272

[82] Li S Li X Xue W Zhang L Yang L-Z Cao S-M. Screening for functional circular RNAs using the CRISPR-Cas13 system. Nat Methods. (2021) 18:51–9. doi: 10.1038/s41592-020-01011-4 33288960

