# Supplementary material for: circ-0001875 downregulation is associated with M1 macrophage activation and lung inflammation in severe asthma
Source: Front Immunol. 2025 Jun 30;16:1601272. doi: 10.3389/fimmu.2025.1601272 (PMC12256210; doi:10.3389/fimmu.2025.1601272)
Supplement: Supplementary file 1 [file DataSheet1.docx]

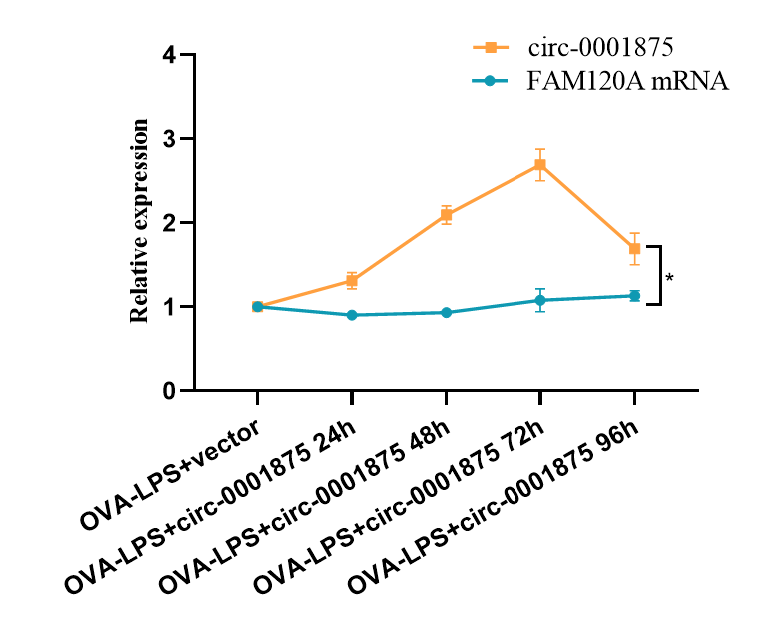


Figure S1 Expression of circ-0001875 and FAM120A in mouse lung tissue after plasmid transfection for 24-96 hours.


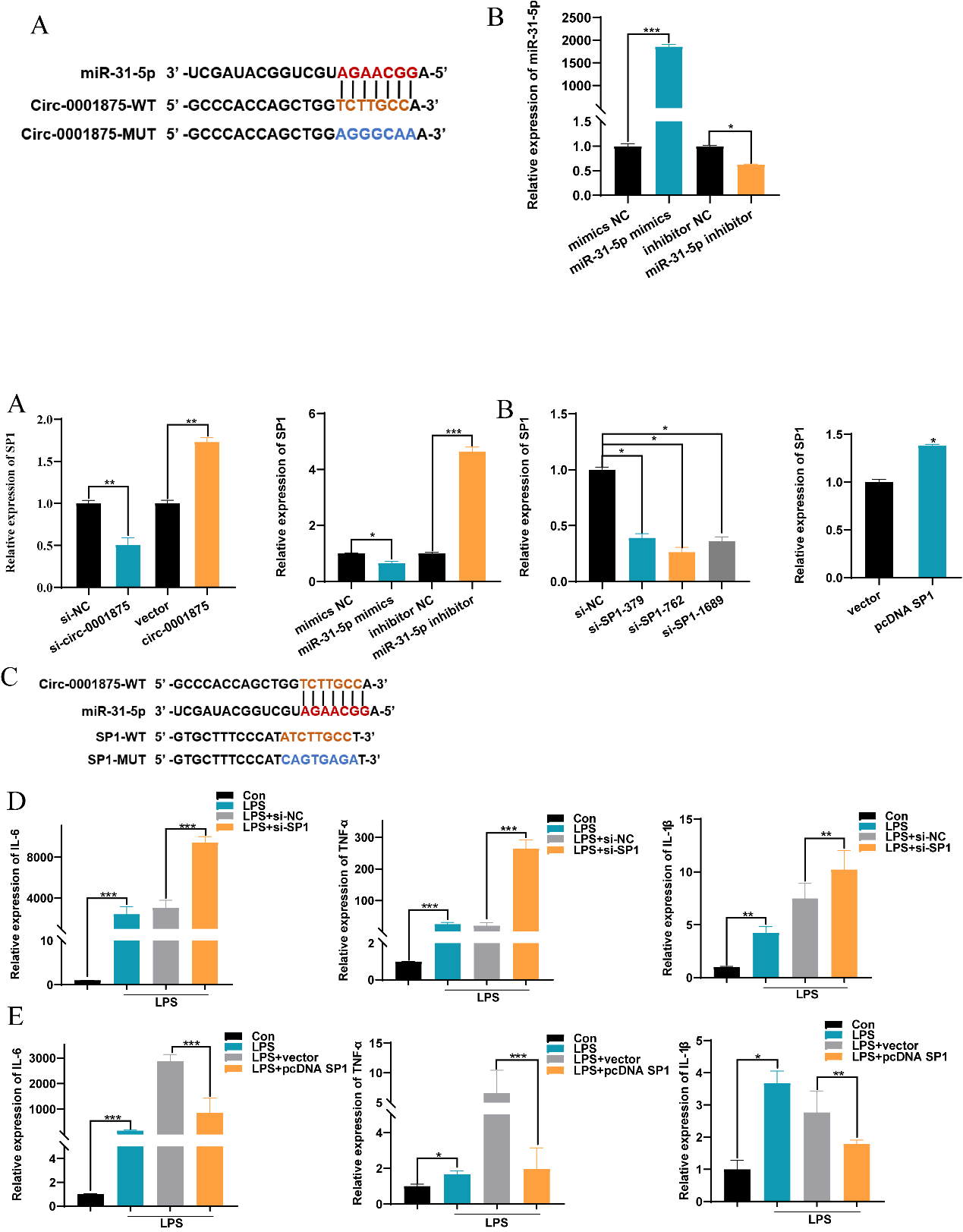


Figure S2 (A) Schematic of the luciferase reporter plasmids for wild-type (circ-0001875 WT) and mutant (circ-0001875 MUT) circ-0001875. (B) Relative expression of miR-31-5p in THP1 cells transfected with miR-31-5p mimics and an miR-31-5p inhibitor, as detected by RT-qPCR.


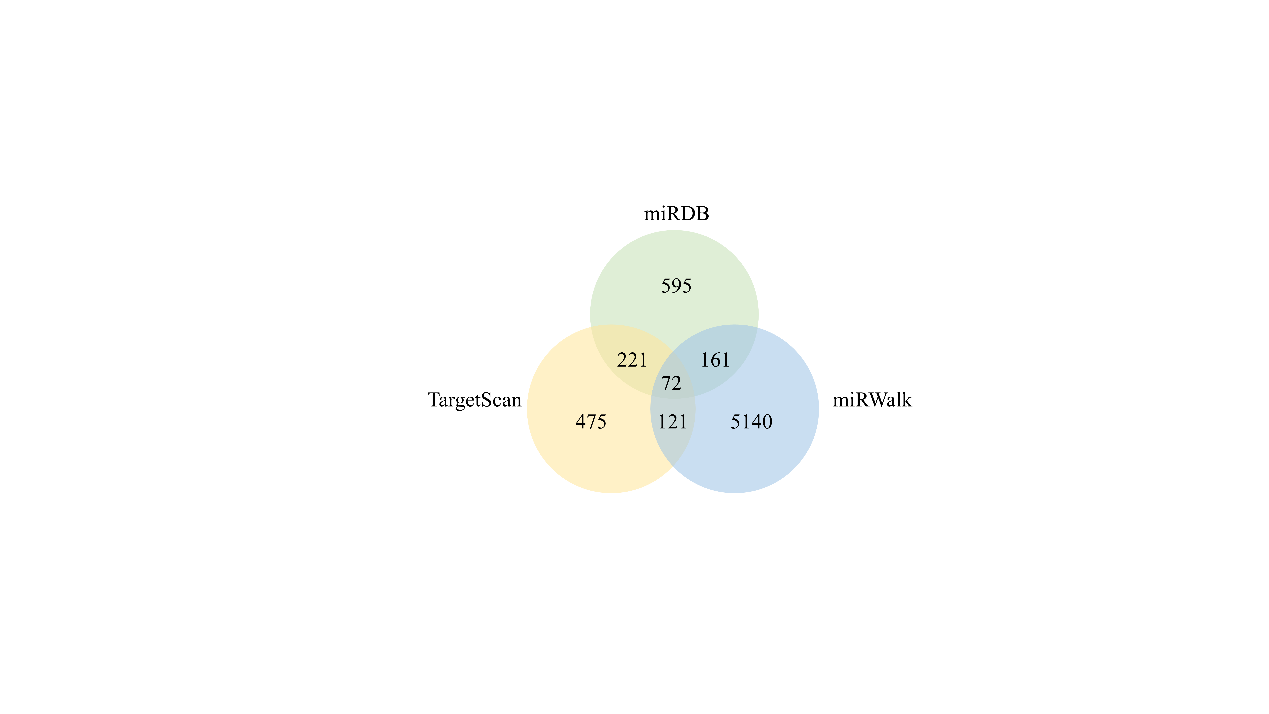


Figure S3 Venn diagram showing 72 genes that are putative miR-31-5p targets predicted by three databases (Targetscan, miRDB and miRWalk).


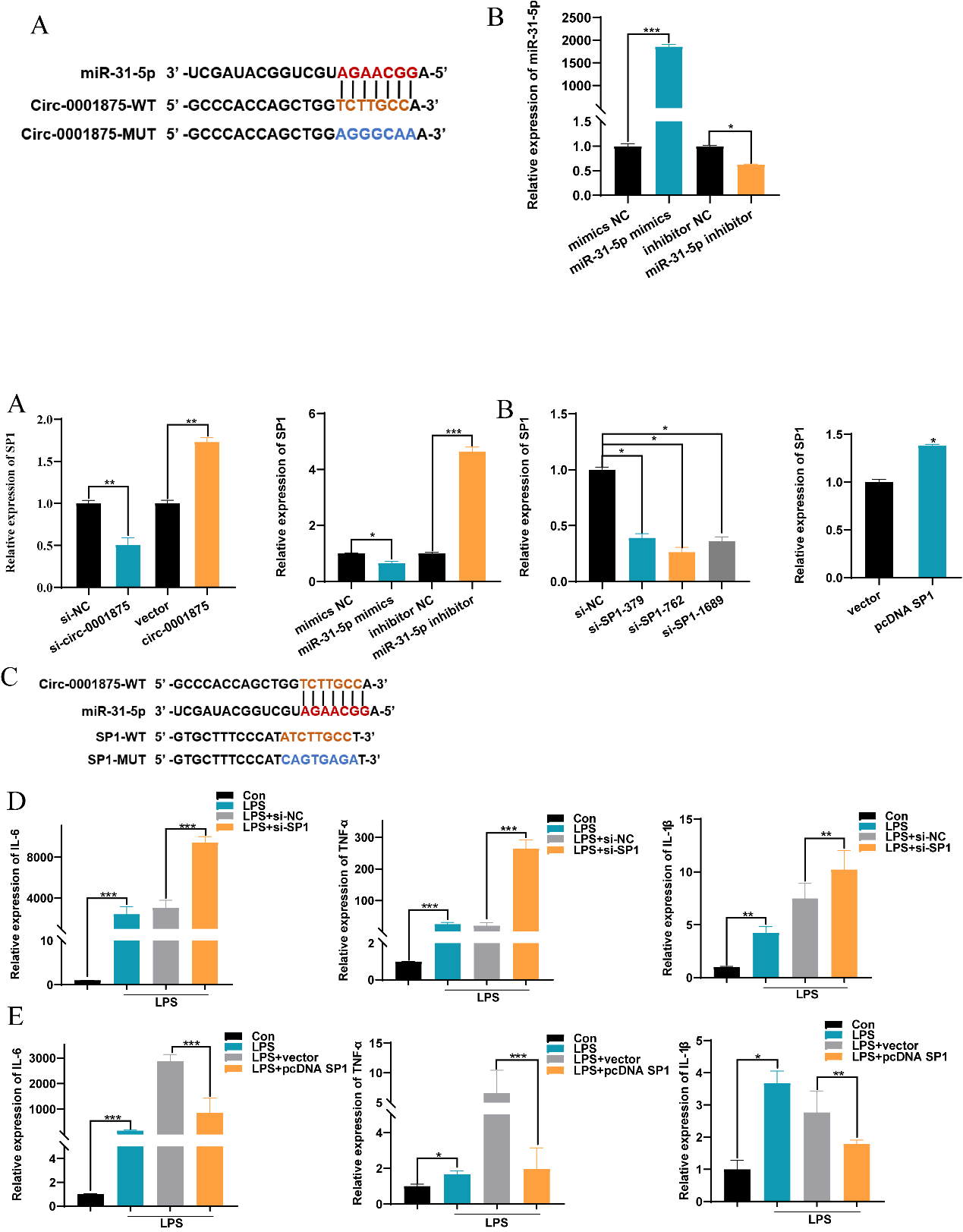


Figure S4 (A) THP1 cells were transfected with circ-0001875 knockdown or overexpression plasmids and miR-31-5p mimics or an inhibitor, and relative SP1 expression was detected by RT-qPCR. (B) SP1 mRNA and protein expression levels in THP1 cells were measured after SP1 knockdown (si-SP1) or overexpression (pc-SP1). (C) Schematic of the wild-type (SP1 WT) and mutant (SP1 MUT) SP1 luciferase reporter plasmids. (D) The effect of si-SP1 on macrophage polarization was detected by RT-qPCR. (E).The effect of pc-SP1 on macrophage polarization was detected by RT-qPCR.


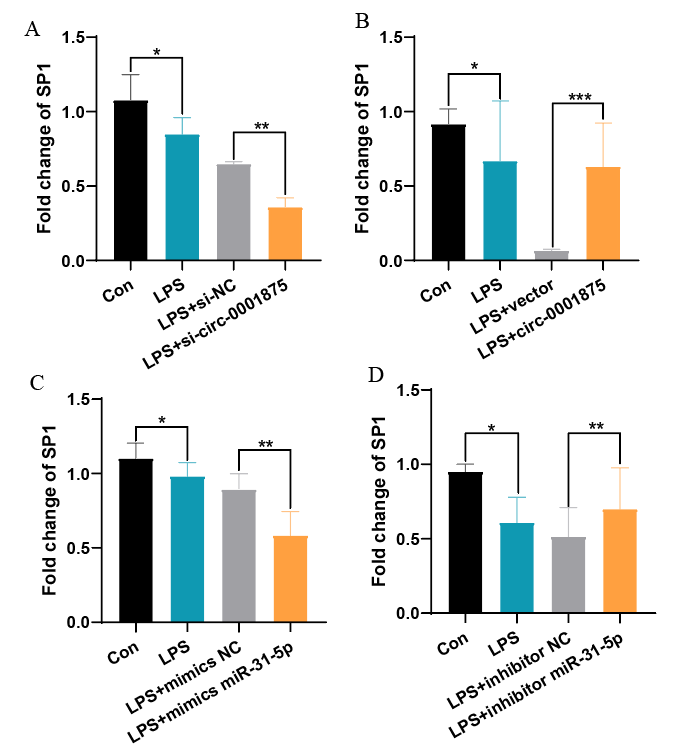


Figure S5 (A-B) THP1 cells were transfected with circ-0001875 knockdown or overexpression plasmids, and relative SP1 expression was detected by Western blot. (C-D) THP1 cells were transfected with miR-31-5p mimics or an inhibitor, and relative SP1 expression was detected by Western blot.
