# Supplementary material for: circ-0001875 downregulation is associated with M1 macrophage activation and lung inflammation in severe asthma
Source: Front Immunol. 2025 Jun 30;16:1601272. doi: 10.3389/fimmu.2025.1601272 (PMC12256210; doi:10.3389/fimmu.2025.1601272)
Supplement: Supplementary file 2 [file DataSheet2.docx]

**Table S1.** **Different expressed circRNAs between severe asthma and healthy control.**

| circRNA | Log_2_FoldChange | *P* value | Up/Down |
| --- | --- | --- | --- |
| hsa_circ_0011362 | -5.097131467 | 0.001016753 | down |
| hsa_circ_0015837 | -5.016393361 | 0.003419599 | down |
| hsa_circ_0001875 | -5.012178922 | 0.001019437 | down |
| hsa_circ_0005925 | -5.011543798 | 0.00180311 | down |
| hsa_circ_0001668 | -5.000148219 | 0.001832842 | down |
| hsa_circ_0031047 | -4.995309498 | 0.00142016 | down |
| hsa_circ_0017236 | -4.943223535 | 0.001826729 | down |
| hsa_circ_0023947 | -4.943038819 | 0.001530678 | down |
| hsa_circ_0032514 | -4.940063633 | 0.002041871 | down |
| hsa_circ_0008294 | -4.903696387 | 0.001683671 | down |
| hsa_circ_0038085 | -4.899597638 | 0.002405721 | down |
| hsa_circ_0016267 | -4.863689117 | 0.003052375 | down |
| hsa_circ_0039427 | -4.851562247 | 0.007681591 | down |
| hsa_circ_0025584 | -4.782008917 | 0.005322905 | down |
| hsa_circ_0030544 | -4.76040216 | 0.011205537 | down |
| hsa_circ_0020663 | -4.745337783 | 0.003519758 | down |
| hsa_circ_0019276 | -4.691704944 | 0.01028617 | down |
| hsa_circ_0033223 | -4.69106872 | 0.004758354 | down |
| hsa_circ_0039936 | -4.68485956 | 0.013190037 | down |
| hsa_circ_0037342 | -4.674073486 | 0.004194532 | down |
| hsa_circ_0023725 | -4.666002342 | 0.00592768 | down |
| hsa_circ_0014651 | -4.645918041 | 0.011937985 | down |
| hsa_circ_0035633 | -4.627389342 | 0.005896767 | down |
| hsa_circ_0009753 | -4.614625456 | 0.006085236 | down |
| hsa_circ_0034149 | -4.607119155 | 0.006194288 | down |
| hsa_circ_0033049 | -4.605492541 | 0.015419737 | down |
| hsa_circ_0012483 | -4.598387039 | 0.005821647 | down |
| hsa_circ_0009491 | -4.561282156 | 0.017289533 | down |
| hsa_circ_0021824 | -4.560530997 | 0.006983875 | down |
| hsa_circ_0009913 | -4.530810813 | 0.023453066 | down |
| hsa_circ_0016754 | -4.529521273 | 0.02151407 | down |
| hsa_circ_0000188 | -4.524578997 | 0.019136642 | down |
| hsa_circ_0028112 | -4.52063314 | 0.007251171 | down |
| hsa_circ_0016526 | -4.507983592 | 0.018618898 | down |
| hsa_circ_0011158 | -4.460638918 | 0.018290761 | down |
| hsa_circ_0043280 | -4.457575562 | 0.018356405 | down |
| hsa_circ_0043300 | -4.454770189 | 0.022010982 | down |
| hsa_circ_0022862 | -4.454270169 | 0.012409849 | down |
| hsa_circ_0021176 | -4.453364195 | 0.020714727 | down |
| hsa_circ_0042829 | -4.449006126 | 0.020007389 | down |
| hsa_circ_0041289 | -4.411187749 | 0.010463295 | down |
| hsa_circ_0040461 | -4.406827053 | 0.020932932 | down |
| hsa_circ_0014867 | -4.401302025 | 0.027958288 | down |
| hsa_circ_0021861 | -4.399134619 | 0.024663236 | down |
| hsa_circ_0043296 | -4.398568403 | 0.024087791 | down |
| hsa_circ_0022554 | -4.396472041 | 0.025498598 | down |
| hsa_circ_0013803 | -4.392017004 | 0.049951383 | down |
| hsa_circ_0003415 | -4.39109528 | 0.011276771 | down |
| hsa_circ_0030345 | -4.388076453 | 0.02829466 | down |
| hsa_circ_0038243 | -4.385294047 | 0.012267922 | down |
| hsa_circ_0028714 | -4.371436626 | 0.04505836 | down |
| hsa_circ_0043288 | -4.368926864 | 0.014537539 | down |
| hsa_circ_0000289 | -4.366659849 | 0.012767746 | down |
| hsa_circ_0010855 | -4.356465319 | 0.012702943 | down |
| hsa_circ_0026528 | -4.337186518 | 0.02567504 | down |
| hsa_circ_0026528 | -4.337186518 | 0.02567504 | down |
| hsa_circ_0025868 | -4.322018808 | 0.0185884 | down |
| hsa_circ_0024395 | -4.309235625 | 0.031728902 | down |
| hsa_circ_0024395 | -4.309235625 | 0.031728902 | down |
| hsa_circ_0036963 | -4.301353998 | 0.029568435 | down |
| hsa_circ_0018664 | -4.295323045 | 0.014709238 | down |
| hsa_circ_0020083 | -4.291554384 | 0.032190726 | down |
| hsa_circ_0042679 | -4.286029685 | 0.029178207 | down |
| hsa_circ_0025655 | -4.280584967 | 0.035003044 | down |
| hsa_circ_0017225 | -4.276799243 | 0.016303997 | down |
| hsa_circ_0010171 | -4.275238432 | 0.016511527 | down |
| hsa_circ_0001008 | -4.271737903 | 0.034566496 | down |
| hsa_circ_0008160 | -4.268222942 | 0.033389263 | down |
| hsa_circ_0023592 | -4.26645151 | 0.031943598 | down |
| hsa_circ_0004987 | -4.264880778 | 0.016319453 | down |
| hsa_circ_0009238 | -4.264796787 | 0.019147062 | down |
| hsa_circ_0001337 | -4.264789916 | 0.017437404 | down |
| hsa_circ_0023962 | -4.261356145 | 0.030863167 | down |
| hsa_circ_0021738 | -4.259396742 | 0.030901225 | down |
| hsa_circ_0010791 | -4.25750019 | 0.01625965 | down |
| hsa_circ_0000507 | -4.256866586 | 0.018002874 | down |
| hsa_circ_0032816 | -4.254879718 | 0.035001424 | down |
| hsa_circ_0032816 | -4.254879718 | 0.035001424 | down |
| hsa_circ_0035354 | -4.245412164 | 0.036541581 | down |
| hsa_circ_0017370 | -4.234249856 | 0.017984549 | down |
| hsa_circ_0025199 | -4.232688283 | 0.034985852 | down |
| hsa_circ_0004028 | -4.229836047 | 0.017886966 | down |
| hsa_circ_0004082 | -4.226540692 | 0.034455775 | down |
| hsa_circ_0026604 | -4.222977462 | 0.03391172 | down |
| hsa_circ_0009880 | -4.221308732 | 0.019814231 | down |
| hsa_circ_0014449 | -4.218642088 | 0.033484225 | down |
| hsa_circ_0031679 | -4.217106955 | 0.035268744 | down |
| hsa_circ_0038329 | -4.210695121 | 0.019473841 | down |
| hsa_circ_0038329 | -4.210695121 | 0.019473841 | down |
| hsa_circ_0000210 | -4.208616319 | 0.03982375 | down |
| hsa_circ_0040851 | -4.206436774 | 0.037894435 | down |
| hsa_circ_0025313 | -4.203929943 | 0.035091957 | down |
| hsa_circ_0038588 | -4.203605246 | 0.034613137 | down |
| hsa_circ_0000817 | -4.200457432 | 0.041496961 | down |
| hsa_circ_0008829 | -4.200426343 | 0.039136675 | down |
| hsa_circ_0011064 | -4.199986954 | 0.038639098 | down |
| hsa_circ_0029489 | -4.198056603 | 0.039244353 | down |
| hsa_circ_0023358 | -4.197292511 | 0.03853341 | down |
| hsa_circ_0030050 | -4.191807242 | 0.037650294 | down |
| hsa_circ_0034122 | -4.188535424 | 0.038811292 | down |
| hsa_circ_0009061 | -4.186739399 | 0.038093026 | down |
| hsa_circ_0027215 | -4.179858633 | 0.024307533 | down |
| hsa_circ_0013246 | -4.171354832 | 0.03725009 | down |
| hsa_circ_0010187 | -4.171050313 | 0.021365294 | down |
| hsa_circ_0015211 | -4.16875674 | 0.02664314 | down |
| hsa_circ_0034530 | -4.166276568 | 0.040872744 | down |
| hsa_circ_0039103 | -4.165074555 | 0.04691716 | down |
| hsa_circ_0036826 | -4.160361931 | 0.038795601 | down |
| hsa_circ_0020387 | -4.157393342 | 0.040974733 | down |
| hsa_circ_0003363 | -4.15595731 | 0.038848571 | down |
| hsa_circ_0034494 | -4.154416105 | 0.026868263 | down |
| hsa_circ_0007753 | -4.144751784 | 0.039805563 | down |
| hsa_circ_0008302 | -4.144107599 | 0.042926942 | down |
| hsa_circ_0003340 | -4.136443349 | 0.045868669 | down |
| hsa_circ_0023918 | -4.134538166 | 0.045047221 | down |
| hsa_circ_0028491 | -4.131770688 | 0.024236727 | down |
| hsa_circ_0001976 | -4.130303015 | 0.041973666 | down |
| hsa_circ_0027461 | -4.128329219 | 0.046028435 | down |
| hsa_circ_0015152 | -4.123147922 | 0.048615406 | down |
| hsa_circ_0002857 | -4.12192824 | 0.023976321 | down |
| hsa_circ_0041871 | -4.118305971 | 0.026449504 | down |
| hsa_circ_0031381 | -4.114499331 | 0.025335867 | down |
| hsa_circ_0022108 | -4.112505321 | 0.049867486 | down |
| hsa_circ_0003326 | -4.110776722 | 0.049049162 | down |
| hsa_circ_0035938 | -4.108129229 | 0.031708982 | down |
| hsa_circ_0026703 | -4.108000077 | 0.043271009 | down |
| hsa_circ_0013649 | -4.103750186 | 0.046788863 | down |
| hsa_circ_0031307 | -4.101249716 | 0.02971807 | down |
| hsa_circ_0008148 | -4.091725011 | 0.028126292 | down |
| hsa_circ_0006286 | -4.088921244 | 0.044834821 | down |
| hsa_circ_0038104 | -4.0848401 | 0.045279605 | down |
| hsa_circ_0020888 | -4.084719291 | 0.027743471 | down |
| hsa_circ_0012975 | -4.084261837 | 0.0309638 | down |
| hsa_circ_0040289 | -4.077201652 | 0.035586077 | down |
| hsa_circ_0033677 | -4.076031376 | 0.027474956 | down |
| hsa_circ_0041618 | -4.07510591 | 0.048928491 | down |
| hsa_circ_0042935 | -4.067773239 | 0.029730175 | down |
| hsa_circ_0008019 | -4.063129852 | 0.047191804 | down |
| hsa_circ_0014990 | -4.055461693 | 0.04834214 | down |
| hsa_circ_0032486 | -4.046406176 | 0.049894365 | down |
| hsa_circ_0032486 | -4.046406176 | 0.049894365 | down |
| hsa_circ_0000178 | -4.037742934 | 0.049833516 | down |
| hsa_circ_0009386 | -4.03441546 | 0.032896137 | down |
| hsa_circ_0022468 | -4.030289119 | 0.031941796 | down |
| hsa_circ_0022468 | -4.030289119 | 0.031941796 | down |
| hsa_circ_0022468 | -4.030289119 | 0.031941796 | down |
| hsa_circ_0015222 | -4.009368029 | 0.034406913 | down |
| hsa_circ_0015222 | -4.009368029 | 0.034406913 | down |
| hsa_circ_0027522 | -4.00838552 | 0.033302989 | down |
| hsa_circ_0037846 | -3.977862225 | 0.037395298 | down |
| hsa_circ_0021553 | -3.976473994 | 0.035856456 | down |
| hsa_circ_0033715 | -3.969592466 | 0.036653661 | down |
| hsa_circ_0039579 | -3.959079629 | 0.038425014 | down |
| hsa_circ_0027009 | -3.953588881 | 0.037659968 | down |
| hsa_circ_0038963 | -3.942930469 | 0.040574635 | down |
| hsa_circ_0014347 | -3.939374698 | 0.039459913 | down |
| hsa_circ_0016538 | -3.91453414 | 0.043117147 | down |
| hsa_circ_0016538 | -3.91453414 | 0.043117147 | down |
| hsa_circ_0018003 | -3.897952112 | 0.047467456 | down |
| hsa_circ_0033342 | -3.897947943 | 0.045875273 | down |
| hsa_circ_0038754 | -3.894550689 | 0.006044034 | down |
| hsa_circ_0042118 | -3.893004262 | 0.044944131 | down |
| hsa_circ_0038546 | -3.892716965 | 0.044894455 | down |
| hsa_circ_0038546 | -3.892716965 | 0.044894455 | down |
| hsa_circ_0025858 | -3.891349881 | 0.043093186 | down |
| hsa_circ_0029645 | -3.886053283 | 0.047262325 | down |
| hsa_circ_0006975 | -3.880469761 | 0.04924343 | down |
| hsa_circ_0022270 | -3.866055065 | 0.04923696 | down |
| hsa_circ_0025151 | -3.842337439 | 0.048841234 | down |
| hsa_circ_0023239 | -3.435965131 | 0.01138001 | down |
| hsa_circ_0012266 | -3.427211021 | 0.020472352 | down |
| hsa_circ_0023587 | -3.403850001 | 0.018154513 | down |
| hsa_circ_0041832 | -3.364177048 | 0.025304934 | down |
| hsa_circ_0030135 | -3.358288302 | 0.026952453 | down |
| hsa_circ_0033486 | -3.346868058 | 0.032916831 | down |
| hsa_circ_0032564 | -3.346825662 | 0.027556337 | down |
| hsa_circ_0022555 | -3.31117572 | 0.005064685 | down |
| hsa_circ_0025235 | -3.273508695 | 0.01213029 | down |
| hsa_circ_0000302 | -3.272480925 | 0.034796752 | down |
| hsa_circ_0012535 | -3.260202053 | 0.038609232 | down |
| hsa_circ_0011513 | -3.247216555 | 0.01746135 | down |
| hsa_circ_0009266 | -3.19708253 | 0.009728082 | down |
| hsa_circ_0034971 | -3.195134946 | 0.009907805 | down |
| hsa_circ_0002656 | -3.191791936 | 0.03808429 | down |
| hsa_circ_0007446 | -3.138348921 | 0.045906001 | down |
| hsa_circ_0015969 | -3.101215869 | 0.028017628 | down |
| hsa_circ_0023074 | -3.068609304 | 0.030319592 | down |
| hsa_circ_0027334 | -3.055361288 | 0.021406888 | down |
| hsa_circ_0017878 | -3.011893051 | 0.0252458 | down |
| hsa_circ_0033352 | -2.99879723 | 0.041227469 | down |
| hsa_circ_0020776 | -2.99506772 | 0.024299123 | down |
| hsa_circ_0016110 | -2.952930638 | 0.048395287 | down |
| hsa_circ_0016110 | -2.952930638 | 0.048395287 | down |
| hsa_circ_0036774 | -2.940057025 | 0.043294722 | down |
| hsa_circ_0003965 | -2.931361753 | 0.045555451 | down |
| hsa_circ_0009169 | -2.8959913 | 0.030168779 | down |
| hsa_circ_0009169 | -2.8959913 | 0.030168779 | down |
| hsa_circ_0019678 | -2.888621167 | 0.0214288 | down |
| hsa_circ_0039513 | -2.864856479 | 0.045260455 | down |
| hsa_circ_0031687 | -2.769350435 | 0.036070365 | down |
| hsa_circ_0001819 | -2.761874223 | 0.031132394 | down |
| hsa_circ_0016637 | -2.756491507 | 0.043920652 | down |
| hsa_circ_0019835 | -2.718964506 | 0.038238748 | down |
| hsa_circ_0040435 | -2.695084918 | 0.040024906 | down |
| hsa_circ_0016835 | -2.661873789 | 0.032321479 | down |
| hsa_circ_0022126 | -2.657404532 | 0.014100503 | down |
| hsa_circ_0024557 | -2.635819559 | 0.035411367 | down |
| hsa_circ_0006737 | -2.61592095 | 0.029580754 | down |
| hsa_circ_0017309 | -2.604061736 | 0.004857062 | down |
| hsa_circ_0015440 | -2.484362501 | 0.016450063 | down |
| hsa_circ_0034866 | -2.446345921 | 0.034007027 | down |
| hsa_circ_0020894 | -2.428393055 | 0.027286371 | down |
| hsa_circ_0019667 | -2.290608909 | 0.039428725 | down |
| hsa_circ_0042186 | -2.262359946 | 0.006109408 | down |
| hsa_circ_0020265 | -2.218379078 | 0.017130567 | down |
| hsa_circ_0019434 | -2.152271521 | 0.035914344 | down |
| hsa_circ_0033046 | -1.955946348 | 0.024004879 | down |
| hsa_circ_0015963 | -1.884267007 | 0.048370814 | down |
| hsa_circ_0026844 | -1.883039127 | 0.016751907 | down |
| hsa_circ_0006265 | -1.873944627 | 0.046137054 | down |
| hsa_circ_0033394 | -1.873207255 | 0.03156761 | down |
| hsa_circ_0000230 | -1.797396434 | 0.029321571 | down |
| hsa_circ_0004002 | -1.779459482 | 0.012615752 | down |
| hsa_circ_0024316 | -1.772585626 | 0.022687615 | down |
| hsa_circ_0023524 | -1.615372317 | 0.03567645 | down |
| hsa_circ_0034251 | -1.418438394 | 0.020535373 | down |
| hsa_circ_0000075 | -1.383511863 | 0.036211919 | down |
| hsa_circ_0020177 | -1.285476518 | 0.031516425 | down |
| hsa_circ_0038786 | -1.232970374 | 0.036704382 | down |
| hsa_circ_0002335 | -1.201133461 | 0.049498536 | down |
| hsa_circ_0013174 | -1.172198266 | 0.004800844 | down |
| hsa_circ_0041546 | -1.148427802 | 0.03581769 | down |
| hsa_circ_0014432 | -1.026172716 | 0.048956783 | down |
| hsa_circ_0043662 | 7.761719632 | 0.021995216 | up |
| hsa_circ_0014664 | 7.419692446 | 0.028626 | up |
| hsa_circ_0037878 | 7.31122876 | 0.03106256 | up |
| hsa_circ_0026014 | 6.931824159 | 0.041046581 | up |
| hsa_circ_0023027 | 6.364548301 | 0.000199456 | up |
| hsa_circ_0023027 | 6.364548301 | 0.000199456 | up |
| hsa_circ_0041508 | 5.876736297 | 0.001148613 | up |
| hsa_circ_0010121 | 5.804969218 | 0.001446449 | up |
| hsa_circ_0026576 | 5.732901859 | 0.001958863 | up |
| hsa_circ_0033873 | 5.692049704 | 0.002523242 | up |
| hsa_circ_0017086 | 5.654492124 | 0.007201685 | up |
| hsa_circ_0027186 | 5.635802156 | 0.003699769 | up |
| hsa_circ_0033899 | 5.593239268 | 0.00858273 | up |
| hsa_circ_0031575 | 5.592264625 | 0.008672417 | up |
| hsa_circ_0023081 | 5.569870942 | 0.004346015 | up |
| hsa_circ_0038198 | 5.529306027 | 0.02180429 | up |
| hsa_circ_0028314 | 5.525417158 | 0.004898491 | up |
| hsa_circ_0000056 | 5.523517653 | 0.010044783 | up |
| hsa_circ_0008234 | 5.510446916 | 0.010614333 | up |
| hsa_circ_0007484 | 5.487266359 | 0.004760845 | up |
| hsa_circ_0030966 | 5.482036667 | 0.02420361 | up |
| hsa_circ_0043595 | 5.482036667 | 0.02420361 | up |
| hsa_circ_0014493 | 5.467729318 | 0.012501914 | up |
| hsa_circ_0007937 | 5.462794958 | 0.025276284 | up |
| hsa_circ_0007937 | 5.462794958 | 0.025276284 | up |
| hsa_circ_0038926 | 5.460761673 | 0.012696791 | up |
| hsa_circ_0027267 | 5.456246564 | 0.012650319 | up |
| hsa_circ_0017119 | 5.442582351 | 0.012911622 | up |
| hsa_circ_0018520 | 5.436832772 | 0.013779673 | up |
| hsa_circ_0020661 | 5.412936237 | 0.007179164 | up |
| hsa_circ_0031609 | 5.393007059 | 0.029379182 | up |
| hsa_circ_0043082 | 5.375249051 | 0.01545744 | up |
| hsa_circ_0031500 | 5.374566826 | 0.015875712 | up |
| hsa_circ_0010095 | 5.370286147 | 0.007386803 | up |
| hsa_circ_0000700 | 5.350289697 | 0.016136909 | up |
| hsa_circ_0003750 | 5.34378682 | 0.007862981 | up |
| hsa_circ_0021215 | 5.343306863 | 0.017078998 | up |
| hsa_circ_0038998 | 5.31717031 | 0.008862856 | up |
| hsa_circ_0020203 | 5.316280642 | 0.008851285 | up |
| hsa_circ_0009598 | 5.305850516 | 0.018124065 | up |
| hsa_circ_0009598 | 5.305850516 | 0.018124065 | up |
| hsa_circ_0039650 | 5.297773828 | 0.019119954 | up |
| hsa_circ_0011373 | 5.269934511 | 0.03490116 | up |
| hsa_circ_0011373 | 5.269934511 | 0.03490116 | up |
| hsa_circ_0012181 | 5.269934511 | 0.03490116 | up |
| hsa_circ_0012879 | 5.269934511 | 0.03490116 | up |
| hsa_circ_0016168 | 5.269934511 | 0.03490116 | up |
| hsa_circ_0016793 | 5.269934511 | 0.03490116 | up |
| hsa_circ_0022615 | 5.269934511 | 0.03490116 | up |
| hsa_circ_0027960 | 5.269934511 | 0.03490116 | up |
| hsa_circ_0027960 | 5.269934511 | 0.03490116 | up |
| hsa_circ_0029201 | 5.269934511 | 0.03490116 | up |
| hsa_circ_0036446 | 5.269934511 | 0.03490116 | up |
| hsa_circ_0042348 | 5.269934511 | 0.03490116 | up |
| hsa_circ_0042716 | 5.269934511 | 0.03490116 | up |
| hsa_circ_0016180 | 5.249244175 | 0.022691129 | up |
| hsa_circ_0001673 | 5.248086031 | 0.021324612 | up |
| hsa_circ_0029595 | 5.239570472 | 0.011160215 | up |
| hsa_circ_0005071 | 5.224076662 | 0.022554156 | up |
| hsa_circ_0007815 | 5.21586643 | 0.023188858 | up |
| hsa_circ_0039181 | 5.195097781 | 0.043868674 | up |
| hsa_circ_0010712 | 5.173951859 | 0.026426772 | up |
| hsa_circ_0002891 | 5.173853193 | 0.045711433 | up |
| hsa_circ_0041880 | 5.168422306 | 0.025855904 | up |
| hsa_circ_0034579 | 5.166548646 | 0.013847798 | up |
| hsa_circ_0012712 | 5.158508257 | 0.026828775 | up |
| hsa_circ_0037827 | 5.158371634 | 0.026971714 | up |
| hsa_circ_0027243 | 5.156939688 | 0.026472477 | up |
| hsa_circ_0031626 | 5.156939688 | 0.026472477 | up |
| hsa_circ_0022354 | 5.151735419 | 0.026493723 | up |
| hsa_circ_0041344 | 5.143264443 | 0.027307165 | up |
| hsa_circ_0032114 | 5.131463365 | 0.028417636 | up |
| hsa_circ_0003578 | 5.123876969 | 0.027537845 | up |
| hsa_circ_0015691 | 5.110400344 | 0.015945019 | up |
| hsa_circ_0021932 | 5.108403054 | 0.03001344 | up |
| hsa_circ_0021932 | 5.108403054 | 0.03001344 | up |
| hsa_circ_0006223 | 5.098186388 | 0.032041424 | up |
| hsa_circ_0033834 | 5.077026166 | 0.01750778 | up |
| hsa_circ_0017112 | 5.075871369 | 0.032126971 | up |
| hsa_circ_0000341 | 5.070831429 | 0.018156843 | up |
| hsa_circ_0001047 | 5.070285793 | 0.033307056 | up |
| hsa_circ_0001047 | 5.070285793 | 0.033307056 | up |
| hsa_circ_0027010 | 5.066406306 | 0.032361495 | up |
| hsa_circ_0024715 | 5.060597025 | 0.032774612 | up |
| hsa_circ_0007272 | 5.057546208 | 0.033177956 | up |
| hsa_circ_0030696 | 5.057546208 | 0.033177956 | up |
| hsa_circ_0019977 | 5.051053231 | 0.0345043 | up |
| hsa_circ_0020729 | 5.041153081 | 0.021334143 | up |
| hsa_circ_0011223 | 5.038433995 | 0.020676478 | up |
| hsa_circ_0030494 | 5.03446834 | 0.033377012 | up |
| hsa_circ_0022556 | 5.01845987 | 0.02043726 | up |
| hsa_circ_0004869 | 4.987462243 | 0.040383928 | up |
| hsa_circ_0006150 | 4.982588103 | 0.039411083 | up |
| hsa_circ_0011808 | 4.982588103 | 0.039411083 | up |
| hsa_circ_0040510 | 4.976347885 | 0.041407849 | up |
| hsa_circ_0031556 | 4.975623553 | 0.039677255 | up |
| hsa_circ_0012665 | 4.971678735 | 0.025061862 | up |
| hsa_circ_0027887 | 4.959545977 | 0.041890485 | up |
| hsa_circ_0033253 | 4.956297566 | 0.044445107 | up |
| hsa_circ_0019764 | 4.955855932 | 0.043069091 | up |
| hsa_circ_0038054 | 4.953343814 | 0.043269381 | up |
| hsa_circ_0007592 | 4.947135395 | 0.040990388 | up |
| hsa_circ_0026286 | 4.945923174 | 0.043280447 | up |
| hsa_circ_0004034 | 4.943697945 | 0.026012451 | up |
| hsa_circ_0042582 | 4.933225998 | 0.044386739 | up |
| hsa_circ_0036742 | 4.898497806 | 0.04715609 | up |
| hsa_circ_0000305 | 4.87863988 | 0.048838236 | up |
| hsa_circ_0011159 | 4.87863988 | 0.048838236 | up |
| hsa_circ_0020823 | 4.875503479 | 0.04884685 | up |
| hsa_circ_0026100 | 4.872217633 | 0.049069801 | up |
| hsa_circ_0037733 | 4.872217633 | 0.049069801 | up |
| hsa_circ_0016951 | 4.847309539 | 0.03203407 | up |
| hsa_circ_0000590 | 4.83668526 | 0.04991207 | up |
| hsa_circ_0013340 | 4.83668526 | 0.04991207 | up |
| hsa_circ_0014974 | 4.83668526 | 0.04991207 | up |
| hsa_circ_0018234 | 4.777236815 | 0.039304536 | up |
| hsa_circ_0036510 | 4.741374937 | 0.041394568 | up |
| hsa_circ_0024758 | 4.659411466 | 0.048968818 | up |
| hsa_circ_0041689 | 4.599603672 | 0.004700247 | up |
| hsa_circ_0041149 | 4.516913603 | 0.01068763 | up |
| hsa_circ_0014485 | 4.357305252 | 0.000367407 | up |
| hsa_circ_0041724 | 4.049587309 | 0.026139485 | up |
| hsa_circ_0032412 | 4.002900956 | 0.008594872 | up |
| hsa_circ_0028168 | 4.000895965 | 0.047276479 | up |
| hsa_circ_0041946 | 3.984245843 | 0.003280914 | up |
| hsa_circ_0006525 | 3.936659839 | 0.033818272 | up |
| hsa_circ_0025929 | 3.829423339 | 0.033471458 | up |
| hsa_circ_0027101 | 3.806472175 | 0.009094024 | up |
| hsa_circ_0003219 | 3.790653467 | 0.031288541 | up |
| hsa_circ_0015618 | 3.762258226 | 0.029307355 | up |
| hsa_circ_0012339 | 3.738901319 | 0.001123641 | up |
| hsa_circ_0015886 | 3.692604237 | 0.012533422 | up |
| hsa_circ_0002259 | 3.681647901 | 0.024972586 | up |
| hsa_circ_0014529 | 3.651295782 | 0.048346942 | up |
| hsa_circ_0007773 | 3.619210898 | 0.039048478 | up |
| hsa_circ_0007773 | 3.619210898 | 0.039048478 | up |
| hsa_circ_0005178 | 3.569931457 | 0.034469938 | up |
| hsa_circ_0034945 | 3.567162906 | 0.026531917 | up |
| hsa_circ_0022364 | 3.455119109 | 0.041921828 | up |
| hsa_circ_0041709 | 3.454929596 | 0.037236926 | up |
| hsa_circ_0038656 | 3.417929343 | 0.013230793 | up |
| hsa_circ_0037927 | 3.281621533 | 0.002017875 | up |
| hsa_circ_0016501 | 3.204068027 | 0.003068837 | up |
| hsa_circ_0019224 | 3.160240516 | 0.046475159 | up |
| hsa_circ_0003868 | 3.158741776 | 0.005215196 | up |
| hsa_circ_0039300 | 3.075243031 | 0.045213489 | up |
| hsa_circ_0039300 | 3.075243031 | 0.045213489 | up |
| hsa_circ_0021088 | 3.047895434 | 0.047694956 | up |
| hsa_circ_0015627 | 3.030243997 | 0.000563374 | up |
| hsa_circ_0023701 | 3.014744214 | 0.015840482 | up |
| hsa_circ_0004830 | 3.007149404 | 0.03094841 | up |
| hsa_circ_0032411 | 2.999580066 | 0.003550723 | up |
| hsa_circ_0025486 | 2.939920853 | 0.002803323 | up |
| hsa_circ_0018708 | 2.713702603 | 0.041771995 | up |
| hsa_circ_0033476 | 2.545409612 | 0.005412264 | up |
| hsa_circ_0011472 | 2.489796712 | 0.04505727 | up |
| hsa_circ_0024755 | 2.470155841 | 0.020791419 | up |
| hsa_circ_0003751 | 2.429186794 | 0.031140808 | up |
| hsa_circ_0034521 | 2.390147864 | 0.003550381 | up |
| hsa_circ_0016417 | 2.355017612 | 0.00224685 | up |
| hsa_circ_0005621 | 2.34855626 | 0.021455631 | up |
| hsa_circ_0043084 | 2.312845382 | 0.001190976 | up |
| hsa_circ_0023026 | 2.213988622 | 0.009674786 | up |
| hsa_circ_0010572 | 2.101991301 | 0.021821409 | up |
| hsa_circ_0010157 | 2.081914861 | 0.038422652 | up |
| hsa_circ_0010157 | 2.081914861 | 0.038422652 | up |
| hsa_circ_0038947 | 1.9443431 | 0.034895003 | up |
| hsa_circ_0030491 | 1.891179907 | 0.03959232 | up |
| hsa_circ_0002841 | 1.773860016 | 0.037867932 | up |
| hsa_circ_0037214 | 1.766218934 | 0.029137915 | up |
| hsa_circ_0040353 | 1.741367276 | 0.01341346 | up |
| hsa_circ_0040353 | 1.741367276 | 0.01341346 | up |
| hsa_circ_0019359 | 1.734168783 | 0.009463656 | up |
| hsa_circ_0030492 | 1.727057779 | 0.025273474 | up |
| hsa_circ_0041212 | 1.465532053 | 0.029791356 | up |
| hsa_circ_0041507 | 1.454076301 | 0.009990603 | up |
| hsa_circ_0011239 | 1.450911698 | 0.020901947 | up |
| hsa_circ_0037142 | 1.438248864 | 0.012215267 | up |
| hsa_circ_0026455 | 1.427289105 | 0.041410737 | up |
| hsa_circ_0033891 | 1.40543176 | 0.023581837 | up |
| hsa_circ_0021495 | 1.374446021 | 0.006323527 | up |
| hsa_circ_0015751 | 1.327552232 | 0.003156171 | up |
| hsa_circ_0015751 | 1.327552232 | 0.003156171 | up |
| hsa_circ_0019260 | 1.299189757 | 0.039681063 | up |
| hsa_circ_0042022 | 1.286159161 | 0.049736523 | up |
| hsa_circ_0013696 | 1.253930951 | 0.01478318 | up |
| hsa_circ_0015413 | 1.227355665 | 0.019317123 | up |
| hsa_circ_0021610 | 1.219238752 | 0.025011855 | up |
| hsa_circ_0018177 | 1.175518905 | 0.014537754 | up |
| hsa_circ_0025460 | 1.159953108 | 0.026981735 | up |
| hsa_circ_0003165 | 1.153585267 | 0.039461796 | up |
| hsa_circ_0016492 | 1.135433259 | 0.040576692 | up |
| hsa_circ_0030495 | 1.128503666 | 0.040759728 | up |
| hsa_circ_0011474 | 1.097228695 | 0.016878564 | up |
| hsa_circ_0011474 | 1.097228695 | 0.016878564 | up |
| hsa_circ_0015854 | 1.05155134 | 0.032995408 | up |
| hsa_circ_0006019 | 1.016949205 | 0.01240583 | up |

**Table S2. The sequences of primers.**

| Primer | Forward primer (5’-3’) | Reverse primer (5’-3’) |
| --- | --- | --- |
| β-actin | TCTCCCAAGTCCACACAGG | GGCACGAAGGCTCATCA |
| Hsa_circ_0001875 | CCACATTACTTAGGTTGCACAG | CGTTCCGGCTCAGTTTTAGG |
| Hsa_circ_0001875 (convergent) | TACGCAACATTCAGGACACC | GAGTCATACGCAACCAAGCC |
| FAM120A | GATCTGGCTTCCTTTCACTGGA | CCGTTCCGGCTCAGTTTTAGG |
| SP1 | TTTTGATGTGTGGGCTTCT | TGGCTGATGCTCCTTATTG |
| U6 | TGGAACGCTTCACGAATTTGCG | TGGAACGCTTCACGAATTTGCG |
| Hsa-miR-31-5p | TTTTGATGTGTGGGCTTCT | TGGCTGATGCTCCTTATTG |
| IL-6 | GTAGCCGCCCCACACAGA | CATGTCTCCTTTCTCAGGGCT |
| TNF-α | CCCTCCTTCAGACACCCT | GGTTGCCAGCACTTCACT |
| IL-1β | TTGAGTCTGCCCAGTTCC | TTTCTGCTTGAGAGGTGCT |

**Table S3. The sequences of siRNAs.**

| siRNA | Sense (5’-3’) | Antisense (5’-3’) |
| --- | --- | --- |
| Negative control | UUCUCCGAACGUGUCACGUTT | ACGUGACACGUUCGGAGAATT |
| si-circ-0001875-1 | GUUGGAUUCAUUACACCAC | CAACCUAAGUAAUGUGGUG |
| si-circ-0001875-2 | CACAUUACUUAGGUUGCAC | GUGCAACCUAAGUAAUGUG |
| miR-31-5p mimics | AGGCAAGAUGCUGGCAUAGC | CUAUGCCAGCAUCUUGCCUUU |
| inhibitor NC | CAGUACUUUUGUGUAGUACAA | \ |
| miR-31-5p inhibitor | AGCUAUGCCAGCAUCUUGCCU | \ |
| si-SP1-379 | CCUCACAGCCACACAACUUTT | AAGUUGUGUGGCUGUGAGGTT |
| si-SP1-762 | GCAACAUCAUUGCUGCUAUTT | AUAGCAGCAAUGAUGUUGCTT |
| si-SP1-1689 | CCAUUAACCUCAGUGCAUUTT | AAUGCACUGAGGUUAAUGGTT |
